# The role of transcranial magnetic stimulation in understanding attention-related networks in single subjects

**DOI:** 10.1016/j.crneur.2021.100017

**Published:** 2021-07-03

**Authors:** B.E. Yeager, C.C. Dougher, R.H. Cook, J.D. Medaglia

**Affiliations:** a Department of Psychology, Drexel University, Stratton Hall, 3201 Chestnut Street, Philadelphia, PA, 19104, USA; b Department of Neurology, Drexel University College of Medicine, 245 N. 15th Street, Mail Stop 423, New College Building, Suite 7102, Philadelphia, PA, 19102, USA; c Department of Neurology, Perelman School of Medicine, University of Pennsylvania, 3400 Spruce St, Philadelphia, PA, 19104, USA

**Keywords:** Neuromodulation, Personalized neuromodulation, TMS, Attention, Network parcellation, Network neuroscience

## Abstract

Attention is a cognitive mechanism that has been studied through several methodological viewpoints, including animal models, MRI in stroke patients, and fMRI in healthy subjects. Activation-based fMRI research has also pointed to specific networks that activate during attention tasks. Most recently, network neuroscience has been used to study the functional connectivity of large-scale networks for attention to reveal how strongly correlated networks are to each other when engaged in specific behaviors. While neuroimaging has revealed important information about the neural correlates of attention, it is crucial to better understand how these processes are organized and executed in the brain in single subjects to guide theories and treatments for attention. Noninvasive brain stimulation is an effective tool to causally manipulate neural activity to detect the causal roles of circuits in behavior. We describe how combining transcranial magnetic stimulation (TMS) with modern precision network analysis in single-subject neuroimaging could test the roles of regions, circuits, and networks in regulating attention as a pathway to improve treatment effect magnitudes and specificity.

## Highlights

• Though studied for over 100 years, the brain basis of attention is still queried.
• Complexity in frameworks for attention makes brain mapping difficult.
• Relevant brain networks vary significantly across subjects, challenging progress.
• Single-subject neuroimaging with TMS can improve our understanding of attention.

## Graphical abstract

**Figure:**
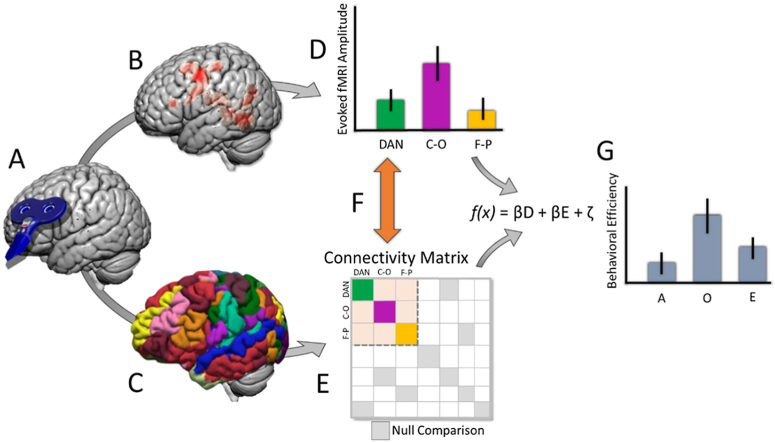

## 1 History of attention

The methods and techniques that are readily available for use in research often directly inform the level at which cognitive phenomena can be understood. With technological advances, our definitions of cognitive phenomena can become more refined and concrete. The cognitive construct of attention has been a vexing concept requiring ongoing refinement. In order to advance our models of attention and its neural basis, converging methods need to be implemented that test competing models using methods that support causal inferences.

At the beginning of the study of attention, solely behavioral observations and anecdotes from researchers and theorists drove the idea of what attention was. William James is considered to be a pioneer for defining attention as “… the taking possession by the mind, in clear and vivid form, of one out of what seem several simultaneously possible objects or trains of thought. Focalization, concentration of consciousness are of its essence. It implies withdrawal from some things to deal effectively with others and is a condition which has a real opposite in the confused, dazed, scatterbrained state …” (James et al., 1890). After James' conceptualization of attention, its definition developed little due to the rise of behaviorism in the early 20th century and the lack of new methodology (Cowan, 1998). However, by the 1950s, behavioral experiments had become dominant in the study of attention with Edward Cherry (1953) and Donald Broadbent (1958) conducting dichotic listening experiments helping to inform theories of selective attention. Then scientists began applying more complex psychophysiological techniques during attention tasks which aided in better understanding how an organism's internal processes play a role in attending to stimuli (Beatty and Kahneman, 1966; Kahneman, 1973; Kahneman et al., 1968, 1969). Simultaneously, behavioral tasks became more complex and measured attention with more nuance and precision. Notably, using these more fine-grained methods, Posner and Boies (1971) were able to suggest that attention is a mechanism that requires three different components: alertness, selectivity among stimuli, and processing capacity. Finally, the latest methodological advancements in animal research and human neuroimaging have built on these behavioral approaches and contributed information about the neural basis of attention processes.

However, the nature of attention mechanisms and their representation in the human brain remains an issue, as the current definitions of attention that we rely on were derived primarily from behavioral research alone. In addition, a persistent challenge in cognitive neuroscience is how to reconcile localizationist (e.g., region-specific) notions of function with the brain's complex network organization (Bassett et al., 2018; Medaglia et al., 2015; Mišić and Sporns, 2016; Sporns, 2014). Notably, a recent emphasis on large scale “intrinsic” networks in the brain has led researchers to ascribe functions to regions that dynamically interact to support cognition and behavior. While these networks are well-established by numerous large-sample studies of the brain at rest and during tasks (Gratton et al., 2018; Kucyi et al., 2018; Power et al., 2011; Yeo et al., 2011), they are fundamentally correlative in nature. Thus, additional methods can help to examine the causal role of brain networks in attention functions. Here, we briefly review the basis of attention from more recent comparative studies of nonhuman primates, neuroimaging in stroke patients, and neuroimaging in healthy people. Then, we clarify the unique role of noninvasive neuromodulation – a means to manipulate neural activity - in revealing *in vivo* principles of attention functions in humans.

### 1.1 Major themes in attention neuroscience

The addition of nonhuman primate, neuroimaging, and noninvasive brain stimulation research has enabled scientists to better understand the neural mechanisms responsible for attention. Though the scope of studies and behavioral findings are vast, we focus here on common major themes in the literature as a foundation to map them onto concepts studied in modern network neuroscience. Particularly, we can organize studies of attention to be focused on top-down/bottom-up processes, endogenous and exogenous orienting, and overt and covert attention. These concepts will be described briefly here while more in-depth reviews of these concepts can be found in the literature.

#### 1.1.1 Top-down and bottom-up processes

Attention mechanisms have been dichotomized into top-down or bottom-up functions, although it's important to point out that instead of being completely exclusive of each other, they may lie on a continuum or even be dependent on one another in particular tasks (Frank and Sabatinelli, 2017; MacLean et al., 2009). Top-down attention is a slower, sustained, effortful, and goal-oriented process involved in the voluntary allocation of attention to stimuli (Baluch and Itti, 2011; Corbetta and Shulman, 2002; Pinto et al., 2013; Ungerleider, 2000). Bottom-up attention is a quicker, transient, automatic, and stimulus-driven process involved in the involuntary allocation of attention to salient or unexpected stimuli (Carrasco, 2011; Corbetta and Shulman, 2002; Pinto et al., 2013; Ungerleider, 2000).

#### 1.1.2 Endogenous and exogenous orienting

Orienting is aligning attention to a particular sensory input or internal structure held in memory (Posner, 1980); in other words, orienting of attention can be modulated through internal (endogenous) or external (exogenous) processes. Endogenous orienting allows the focus of attention to be controlled by task demands, thereby orienting attention to one's goals (Posner, 1980). This process closely reflects the concept of top-down processing, and the nomenclature is often used interchangeably. Conversely, exogenous orienting of attention occurs involuntarily because of unexpected or salient stimuli entering the periphery of the visual field (Berger et al., 2005). Similar to how endogenous orienting is to top-down processing, exogenous attention is analogous to bottom-up processing, due to it being stimulus-cued. Exogenous and endogenous attention are considered independent of one another, like top-down and bottom-up processing, but they interact and are arguably not completely mutually exclusive (Frank and Sabatinelli, 2017; MacLean et al., 2009).

#### 1.1.3 Overt and covert attention

Orienting of attention can also be classified as either overt or covert and can each be driven by top-down or bottom-up mechanisms. Overt orienting of attention involves the physical movement of the head and eyes and shift of gaze to a selective item or location in the visual space (Posner, 1980). Covert attention orienting does not require any eye movements, only a shift of attention driven by internal selection (Klein and Shore, 2000). More specifically, covert attention orienting to stimuli requires one to attend to the extra-foveal region of space without the explicit reorienting of fixation (Kelley et al., 2008; Kulke et al., 2016; Mangun, 1995).

### 1.2 Brain networks and attention

**Fig. 1:**
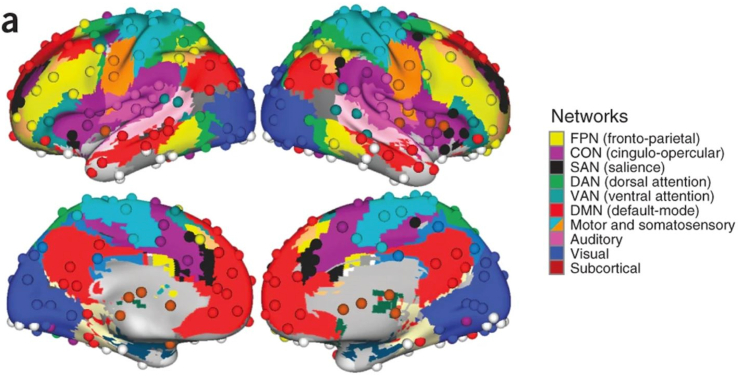
*Network partition of 264 putative functional regions described previously*. The ten major networks (node communities) are labeled on the right. Figure reproduced with permission (Cole et al., 2016).

To date, studies have ascribed behaviors in and out of the attention domain to major intrinsic brain networks. In particular, attention is thought to be rooted in interactions within and between several distinct networks, including the dorsal attention, ventral attention, fronto-parietal control, cingulo-opercular, and default mode networks (see Fig. 1). The primary functions of these networks for the mechanism of attention will be described here.

The dorsal attention network (DAN) is involved in externally directed attentional tasks, including maintaining and guiding visual attention using oculomotor processes and voluntary attention to expected sensory stimuli during goal-directed tasks (Corbetta et al., 2008; Benedek et al., 2016). The ventral attention network (VAN) is involved in internally directed attentional tasks, including detecting salient and behaviorally relevant stimuli and triggering shifts of attention (Corbetta et al., 2008; Vossel et al., 2014). The fronto-parietal control network (FPCN) has been implicated in coordinating behavior in a rapid, accurate, and flexible goal-driven manner, including maintaining and switching set and coordinating activity between networks (Marek and Dosenbach, 2018). The cingulo-opercular network (CO) is often referred to as the “salience” network, which is involved in detecting novel or salient stimuli and maintaining alertness and focus to that stimuli (Uddin, 2016). Finally, the default mode network (DMN) has been shown to be primarily implicated in the disengagement of attention from the external environment and engagement of attention to internal introspective thought (Andrews-Hanna, 2012; Buckner and Carroll, 2007; Raichle, 2010). It has also been thought that the DMN needs to cooperate with the FPCN for efficient conscious internal thought (Smallwood et al., 2012). These distinct lines of evidence reveal complementary data that substantiate the role of these networks as basic “units” that support attention functions.

In this review, we will summarize the major findings that have come from nonhuman primate, stroke neuroimaging, healthy neuroimaging, and noninvasive brain stimulation research. These lines of research have revealed how attention is driven through several networks, including the dorsal attention, ventral attention, frontoparietal control, cingulo-opercular, and default mode networks. We will then discuss how we can most optimally study attention in the future using neuromodulatory techniques paired with subject-specific network analysis and behavioral tasks. Crucially, we will speak to the challenge to reconcile studies of task-evoked activity and intrinsic networks using modern statistical methods paired with TMS.

## 2 Comparative studies in nonhuman primates

Comparative research involving nonhuman primates (NHPs) has offered important insights into basic attention circuits, helping to improve our understanding of attention mechanisms in the brain. NHP models are especially advantageous to use in neuroscience research, as opposed to other animals (i.e., mice, rats), because NHPs are more genetically similar to humans (Vallender and Miller, 2013), have similar overall brain structure and network configuration (Mantini et al., 2013), and have similar cognitive functions as humans (Roelfsema and Treue, 2014). Additionally, a major advantage of NHP research is the use of invasive research techniques that reveal important information about brain anatomy and physiology that cannot ethically be used in humans (Rilling, 2014).

Within comparative research, scientists have been able to use invasive recordings, microstimulation, and neuroimaging techniques to learn more about the neural aspects of attention. In regard to the top-down and bottom-up attention distinction, invasive recordings of neuronal activity in the frontal and parietal cortices of monkeys has helped us to better understand how top-down and bottom-up functions of attention originate from these regions, respectively (Buschman and Miller, 2007). Similarly, a regression-like topological analysis of network connections within the primate cortical visual system revealed that the NHP visual cortex was found to be dichotomized into hierarchically organized dorsal (top-down) and ventral (bottom-up) streams of attention (Young, 1992).

Comparative studies have researched the involvement of neural networks in NHP attention that include the dorsal and ventral attention networks but have extended researchers’ focus to include the frontoparietal control, cingulo-opercular, and default mode networks. The dorsal attention network (DAN) is involved in externally directed attentional tasks, including maintaining and guiding visual attention using oculomotor processes and top-down, voluntary attention to expected sensory stimuli during goal-directed tasks (Benedek et al., 2016; Corbetta et al., 2008). A core anatomical area of the DAN that has been extensively researched in NHPs are the frontal eye fields, which are responsible for saccadic eye movements (Ptak and Schnider, 2010) and shifting attention (Moore and Fallah, 2001). Research applying microstimulation to neurons in the frontal eye fields aimed at enhancing neuronal activity in the extrastriate visual cortex has provided evidence for the involvement of the frontal eye fields in spatial attention distortions (Moore and Armstrong, 2003). Furthermore, research using implanted electrodes in NHP brains has revealed that disruptions to the frontal eye fields inhibit planned attentional saccades but have no effect on reflexive attention (Lee et al., 2012). However, more recent research suggests that the NHP brains may not have as much of a DAN as humans.

The ventral attention network (VAN) is responsible for internally directed attentional tasks, including detecting salient and behaviorally relevant stimuli and triggering shifts of attention (Corbetta et al., 2008; Vossel et al., 2014). The temporoparietal junction (TPJ) in the ventral parietal cortex is considered to be the most prominent feature of the human VAN, and it is involved in the involuntary reorientation of attention to currently unattended stimuli and especially to stimuli that are unexpected or salient (Corbetta and Shulman, 2002; Downar et al., 2000; Serences et al., 2005). A functional connectivity study in monkeys revealed a topological and functional equivalent VAN in macaques as is found in humans (Mantini et al., 2013). However, the results of this study show that the VAN found in monkeys covers more areas than the VAN found in humans and has no overall unifying function (Mantini et al., 2013; Patel et al., 2015); this perhaps suggests a discrepancy between NHPs and humans for VAN architecture and function. Providing additional support to this notion, much of NHP research suggests that there is no homologous brain region or network for the human TPJ specifically, or the human VAN more globally, in NHP models (Constantinidis and Steinmetz, 2001; Orban et al., 2006; Patel et al., 2015). This literature should raise concern for how well NHP network architecture and function can translate to human networks.

In attention, the frontoparietal control network (FPCN) is involved in coordinating behavior in a rapid, accurate, and flexible goal-driven manner, including maintaining and switching set and coordinating activity between networks (Marek and Dosenbach, 2018). This network has been a key topic of NHP research aimed at understanding attentional control. Particularly, the lateral prefrontal cortex is a key area of the FPCN responsible for initiating and modulating cognitive control (Dosenbach et al., 2008) and is a popular area of research in comparative studies. For instance, following the removal of the prefrontal cortex, primates’ ability to switch top-down control was selectively impaired, providing evidence for the involvement of the FPCN in the control of attentive switching (Rossi et al., 2009). Moreover, lesions to the lateral prefrontal cortex in primates have been shown to impair spatial attention (Goldman and Rosvold, 1970). In addition, using single-neuron recordings in primates, the anterior cingulate cortex of the FPCN has been implicated in task maintenance and the implementation of volitional control of attention (Johnston et al., 2007).

The cingulo-opercular network (CO) is involved in detecting novel or salient stimuli and maintaining alertness and focus to that stimuli (Uddin, 2016). Using neuroimaging, an affective salience network rooted in the ventral anterior insula was found in macaque monkeys (Touroutoglou et al., 2016), which corroborates the conception that the anterior insula is a key component to the cingulo-opercular “salience” network (Sadaghiani and D'Esposito, 2015). However, the same research revealed that the connectivity of dorsal anterior insula was not sufficiently developed to form the dorsal salience network (Touroutoglou et al., 2016). These findings do not support research in humans that has identified two dissociable salience networks in humans (Touroutoglou et al., 2012), suggesting the lack of homology between these species.

The default mode network (DMN) has been shown to be implicated in the disengagement of attention from the external environment and engagement of attention to internal introspective thought (Andrews-Hanna, 2012; Buckner and Carroll, 2007; Raichle, 2010) and has been shown to cooperate with the FPCN for efficient conscious internal thought (Smallwood et al., 2012). The posterior cingulate cortex (PCC) is known to play a central role within the DMN and has been verified in NHPs using single neuron recording (Hayden et al., 2009). In addition, using functional neuroimaging with NHPs, research has suggested that there is a network of regions functionally equivalent to the human DMN (Vincent et al., 2007). Another functional neuroimaging study found that the dorsal PCC shows increased functional connectivity with the DMN and shows more anticorrelation with the cognitive control network (FPCN). These results provide evidence that the dorsal PCC plays a role in modulating interactions between the DMN and cognitive control network for the efficient allocation of attention (Leech et al., 2011). However, when comparing the functional similarities of the NHP DMN and human DMN, there was only a small intersubject correlation (Mantini et al., 2013). This result may be explained by the redeployment theory: because of evolutionary demands, certain cortical regions may have been redeployed to be responsible for new functions (Anderson, 2010); in fact, it has been suggested that the DMN may have evolved to support spontaneous cognition (Mantini et al., 2011). The conflicting nature of the DMN research in NHPs should be further considered, as it provides additional support to the previous notion that there may be a distinct lack of homology in the neural architecture and function between NHPs and humans.

The use of invasive neural recordings, microstimulation, and neuroimaging in NHPs has enabled us to find comparative models for attention in the brain and has revealed important information regarding the brain basis of attention functions. Furthermore, NHP research has provided evidence for the notion that there are distinct intrinsic networks for attention in the brain. Though, the variance between NHPs and humans due to methodologies and techniques used and the neuroanatomy of both species is important to note (Cole et al., 2009), and these weaknesses perhaps support the idea for more insightful and controlled attention research to be conducted in humans.

## 3 Neuroimaging research in stroke patients

Aside from comparative studies using invasive probing of neural activity to better understand behavior, noninvasive neuroimaging, such as functional magnetic resonance imaging (fMRI), that provide task activation-based and connectivity-based measures have been used to better understand attention in the brain. Particularly, neuroimaging studies in individuals with stroke have informed basic science because investigators can observe lost function compared to healthy subjects to make inferences about the necessity of brain regions and connections in cognitive functions. Stroke research is often focused on the functional deficits that stroke patients encounter. It should also be noted that much of stroke research has identified specific brain regions implicated in attention function instead of identifying particular networks; however, these brain regions can be related to neural networks as a result of later network-focused research that has linked general brain regions to distinct networks. Here, we will discuss how neuroimaging studies with stroke patients have added to our understanding of the brain basis of attention functions while linking putative brain regions to distinct neural circuitry in the human brain.

Much of the seminal attention research done with stroke patients has corroborated the research done in nonhuman primates, providing some confirmation to those important findings that helped provide a neural basis for attention. Importantly though, this line of research has expanded upon those major findings from comparative research, providing specific information about attention processes in the human brain. For instance, the understanding that attention can be divided into two dichotomous processes that are responsible for controlled and focused attention and involuntary attention has persisted following evidence from stroke research but has also been expanded to involve particular human brain regions. Within this research, the intraparietal cortex and superior frontal cortex have been shown to be primarily responsible for preparing and applying goal-directed selection for stimuli and responses (top-down) and the temporoparietal cortex and inferior frontal cortex have been shown to be responsible for the detection of relevant and salient stimuli (bottom-up) (Corbetta and Shulman, 2002). These brain regions are widely researched in spatial neglect in stroke patients.

As previously noted, stroke research focuses on the deficits that arise following a stroke; one major consequence that can occur that directly impacts attention functions is spatial neglect, in which a reduction of arousal and speed of information processing occurs, thus creating a spatial attention deficit (Corbetta and Shulman, 2011). This disorder provides particularly important information about attention mechanisms because lesions can be correlated with specific deficits in function. It has found that damage to regions including the temporoparietal cortex and inferior frontal cortex lead to deficits in vigilance and reorienting, confirming part of the location of the ventral attention network (VAN) (Corbetta et al., 2005; Corbetta and Shulman, 2002; Husain and Rorden, 2003). Post-stroke, these ventral regions are structurally and functionally disrupted and do not recover (He et al., 2007). In addition, damage from lesions to the parietal cortex near the TPJ causes deficits in reorienting attention toward visual stimuli in unattended locations in patients with neglect (Corbetta et al., 2000). These findings have since been supported by results from another study that found that neglect was associated with damage to the TPJ, middle frontal gyrus, and posterior intraparietal sulcus (Ptak and Schnider, 2011). Moreover, in neglect patients, it has also been found that there is reduced intra-network functional connectivity within the VAN, further implicating this network in attention and perhaps indicating that reduced connectivity is what leads to neglect (Barrett et al., 2019). Overall, these studies identified the role of particular canonical brain regions implicated in the VAN in involuntary spatial attention. Moreover, post stroke ventral lesions that result in neglect can alter the physiology and functioning of other various regions related to attention, specifically within the dorsal frontoparietal regions (Corbetta and Shulman, 2011). The intraparietal sulcus and frontal eye fields are brain regions that have been found to be major parts of the dorsal attention network (DAN), and they have been shown to be largely unaffected by lesions that have caused spatial neglect in patients (Corbetta et al., 2005; Milner and McIntosh, 2005). After VAN damage, these dorsal regions remain structurally sound, however, interhemispheric functional connectivity is disrupted, albeit the dorsal regions do end up fully recovering (He et al., 2007). It is worth noting that the majority of lesions that cause neglect are located in the right hemisphere (Stone et al., 1993; Vallar and Perani, 1986). Importantly, regions of the VAN have also been found to be right lateralized and are the most commonly inflicted regions following these right hemisphere lesions that result in neglect (Corbetta and Shulman, 2002). This right lateralization of the VAN may explain why VAN regions are distinctly affected by lesions while DAN regions and functions are not directly affected by right hemisphere lesions that cause neglect.

Attention has received most of the focus of stroke research because it is believed to be most relevant in spatial neglect, and to this extent, the focus of attention deficits involved in neglect primarily revolve around the VAN and DAN. However, a focus on the role of the other different networks in attention post-stroke, like the frontoparietal control (FPCN), cingulo-opercular (CO), and default mode networks (DMN), is less abundant. However, although not the primary focus, evidence suggests that these networks are involved in the accompanying symptoms of spatial neglect and the overall recovery from stroke. In particular, deficits in conflict resolution, or the ability to adjust behavior in the service of resolving among incompatible representations (Hussey and Novick, 2012), as measured by an attention test, can be found as a result of post-stroke lesions to the bilateral prefrontal areas of the FPCN (Rinne et al., 2013). What's more, increased frontoparietal integration was found to be facilitatory in the recovery of cognitive functioning following stroke and is thought to be a common neural mechanism for increased cognitive control (Sharp et al., 2010). In terms of spatial neglect specifically, more severe neglect was found to be associated with a disconnection of white matter tracts connecting the frontal and parietal cortices (He et al., 2007). Importantly, this research may suggest that the FPCN should be further considered in cases of spatial neglect in addition to the DAN and VAN. It has also been suggested that salience is implicated in attention deficits following stroke (Corbetta and Shulman, 2011), and therefore, the CO should be further studied in cases of stroke. Research has also indicated that the DMN may be relevant to long-term recovery. One study found that DMN connectivity with the contralesional dorsolateral prefrontal cortex had a positive relationship with recovery of cognitive function following a stroke (Park et al., 2014). Therefore, while the DAN and VAN are the extensively studied networks in research of stroke patients and spatial neglect, several other networks, including the FPCN, CO, and DMN may be key networks involved in the symptoms of spatial neglect and recovery of functions. The domain general and specific roles of these functions in attention and spatial neglect post-stroke and recovery remains to be seen.

Because of the nature of post-stroke lesions, stroke research has been important in understanding how particular human brain regions and their connections are implicated in attention. In particular, neuroimaging studies involving patients with spatial neglect have offered influential insights into the role specific brain regions, and thereby specific networks, have in spatial attention. While stroke studies are informative, they are limited as a basic science model due to heterogeneity, neuroplasticity, and the fact that cognitive studies in stroke examine the function of the brain in the context of the lesion, not the function of the region lost due to the stroke. Therefore, although this research has provided vital insight into the roles specific brain regions play in attention following this specific brain injury, it is important to understand how attention is represented in the brain in healthy subjects. Neuroimaging in healthy subjects provides additional information about attention regions, networks, and connections in the brain.

## 4 FMRI research in healthy subjects

**Fig. 2:**
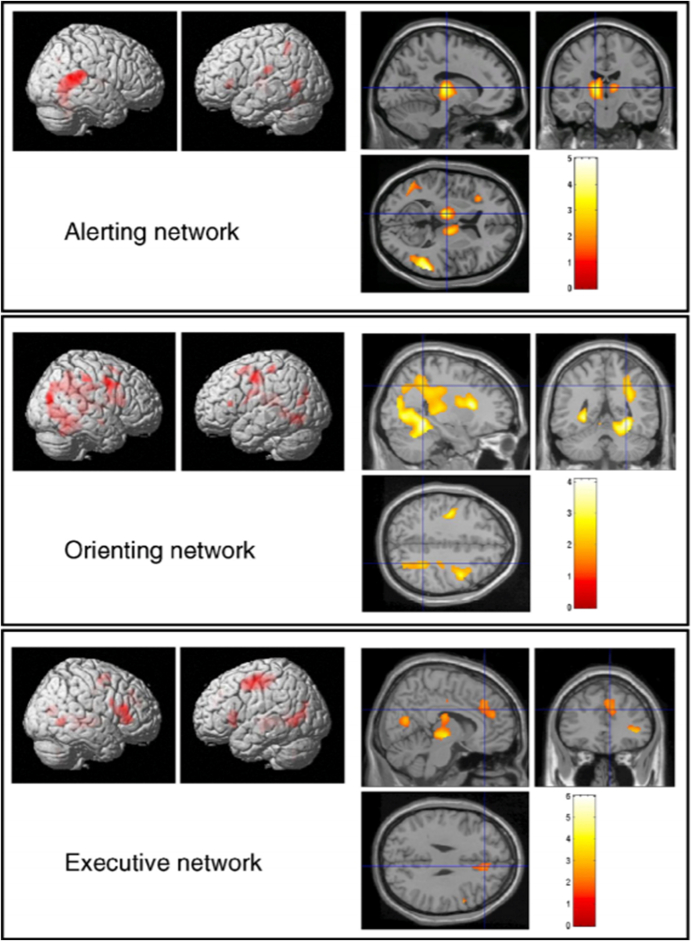
*Event-related fMRI results indicating cortical activation of the three attention networks.* The alerting network view displays activation of the frontoparietal network and thalamus. The orienting network view displays activation of the parietal network. The executive network view displays activation of the anterior cingulate cortex. Figure reproduced with permission (Fan et al., 2005).

Findings from both comparative and stroke research are corroborated within neuroimaging research with healthy subjects, but this line of research also offers more power to identify neural correlates of basic attention. As a result of fMRI studies done in healthy subjects with specified behavioral testing, our understanding of attention has expanded forward from the dichotomization of attention that was revealed from NHP and stroke research. As previously discussed, the NHP visual cortex was found to be dichotomized into hierarchically organized dorsal (top-down) and ventral (bottom-up) streams of attention (Young, 1992). This finding is also true in humans, where a dorsal and ventral stream involved in object vision and spatial vision processing, respectively has been found (Ungerleider and Haxby, 1994). However, in addition to and largely in parallel with MRI stroke research, a dominant viewpoint of human attention networks *in vivo* was established using event-related fMRI activation models in healthy subjects with a highly specific behavioral test. Whereas stroke research placed a major emphasis on regions in the DAN and VAN and focused on the persistent aspects of attention deficits within spatial neglect, healthy human neuroimaging research has instead divided attention into three anatomically distinct subsystems. This research involves cued attention paradigms that heavily focus on the fast, dissociable components of attention, including alerting, orienting, and executive control functions (Posner and Petersen, 1990). In this scheme, alerting can be defined as the ability to respond and maintain vigilance to signals. Orienting is defined as the ability to change the priority of a stimulus by overtly and covertly attending to its new location; this can be done without a change in eye or head movement (Posner, 1988). Executive control of attention can be defined as resolving conflict among stimuli or other mental efforts. Using general linear modeling in fMRI, evidence has indicated separable anatomical substrates for each of these functions of attention (Fan et al., 2005) (see Fig. 2). Cortical activation principally in the frontoparietal network, thalamus, superior colliculus, and right temporal parietal junction have been implicated in alerting. These results coincide with findings from an earlier PET experiment that found activation of the fronto-parietal-thalamic-brainstem network in an alertness task (Sturm et al., 1999). These results point toward the role of the more salience driven attention networks in alerting, like the CO and VAN, which provides some additional support for the results found in stroke research. From the same fMRI research, orienting behavior elicited activation in the left and right superior parietal lobe and the left precentral gyrus. In a different event-related fMRI study, the parietal cortex was shown to control voluntary orienting of attention, in which activation in the intraparietal sulcus and right temporoparietal junction was found (Corbetta et al., 2000). Finally, executive control of attention was linked to activation of the anterior cingulate and left and right frontal areas. Providing further support, results of a separate event-related fMRI study found activation of the anterior cingulate cortex in the presence of conflict (Van Veen and Carter, 2002; Van Veen et al., 2001); these findings further implicate a role for the FPCN in the control of attention.

Although a distinct view by itself, the alerting, orienting, and executive control division of attention found by Posner and Petersen (1990) has been related to the formative ventral and dorsal attention perspective (Petersen and Posner, 2012). This relationship between views has helped corroborate many of the previous findings about the neuroanatomy of attention functions. Specifically, the alerting system is responsible for reacting to warning signals and these signals have been shown to involve activation of the locus coeruleus which is the source of norepinephrine (NE) (Aston-Jones and Cohen, 2005). The NE pathway includes nodes in the frontal and parietal cortices which relate to the dorsal visual pathways (Morrison and Foote, 1986). Additionally, brain regions previously implicated in the orienting system, particularly the intraparietal sulcus/superior parietal lobe, have also been shown to be part of the DAN, in addition to the frontal eye fields (Corbetta and Shulman, 2002), thereby prospectively linking aspects of the subsystem view of three attention networks to the ventral and dorsal attention system perspective.

Although both stroke neuroimaging and healthy subject neuroimaging research have elaborated on our understanding of the neural aspects of attention that appear consistent with a network-level framework for attention functions, it is incomplete. For instance, activation and stroke-based studies tend to be fundamentally localizationist – they ascribe specific functions and dysfunctions to individual regions of the brain. A comprehensive theory for attention processes will additionally need to identify the appropriate levels of network organization that support attention. Ideally, this framework will be consistent with activation-based and lesion studies but extend beyond them to describe and predict the causes of attention functions. To this end, network neuroscience is an appealing domain of inquiry.

### 4.1 The role of network neuroscience in identifying candidate attention networks

**Fig. 3:**
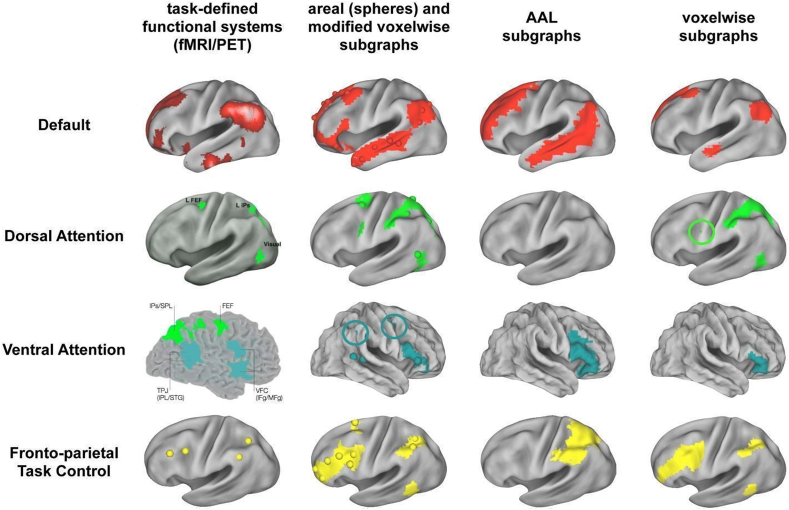
*Graph Definition Dictates Fidelity to Functional Brain Organization.* At left, the task-defined locations of four established functional systems. The next three columns display, for the main cohort, the single subgraph that best corresponds to each functional system under the four graph definitions. Circles are placed around small portions of subgraphs that might otherwise be overlooked. Caption and figure reproduced with permission (Power et al., 2011).

Prior studies have primarily looked at the relative activation of systems during active attention, but recently, evidence has convincingly suggested that there are several major intrinsic networks in the brain present during task and resting conditions. Complementing comparative, stroke, and task activation literature supporting models of attention, a more general viewpoint of cognitive systems relies on network neuroscience to study functional connectivity to reveal the functional network organization of the human brain (Bassett and Sporns, 2017). With these methods, researchers can reveal how large-scale networks are functionally connected to one another. This temporal co-activation of networks can reveal how strongly correlated networks are to each other when at rest or engaged in specific thoughts or behavior (Medaglia et al., 2015). These networks organize functional activation during cognitive tasks (Cole et al., 2016) (see Fig. 3). In terms of reproducibility, networks derived from task-based functional connectivity have been found to be reproducible within brain states, but interestingly, these networks can also reconfigure across various brain states, suggesting that there may be a state-dependent reorganization of functional areas (Salehi et al., 2020a). Furthermore, there is reproducibility for resting-state functional networks as well. Over a 3.5-year period, in which weekly fMRI scans were collected, researchers identified 14 resting state networks whose network spatial maps, temporal signal fluctuations, and between-network connectivity reproducibility was high (Choe et al., 2015).

**Fig. 4:**
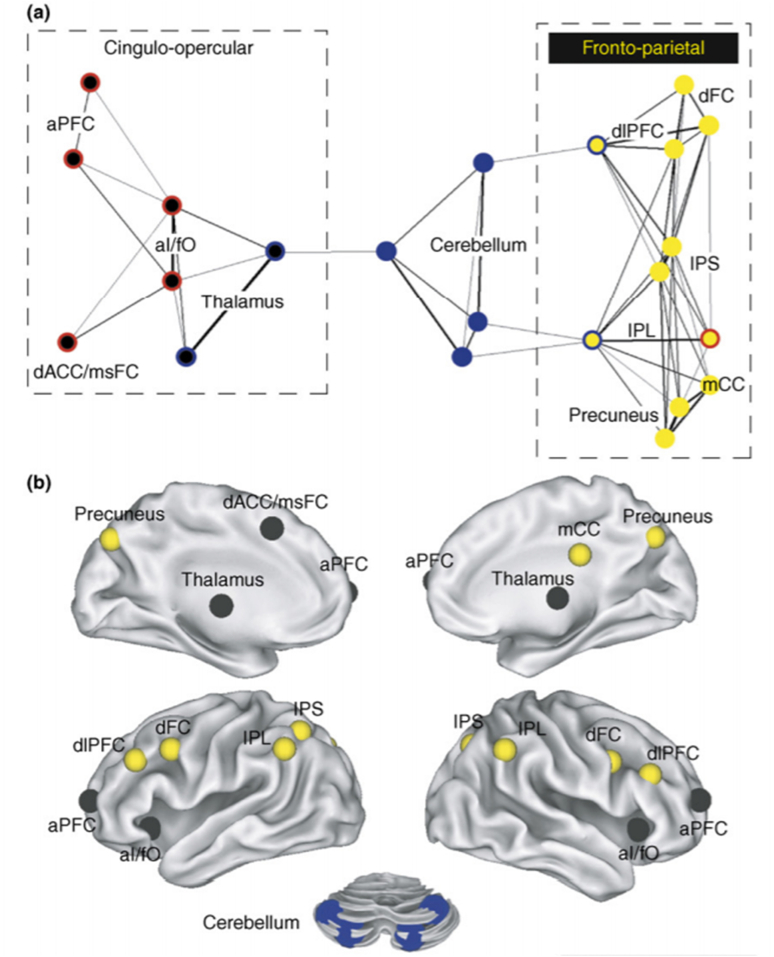
*Distinct frontoparietal and cingulo-opercular control networks.* (a) The network structure of human control networks is displayed in a two-dimensional graph layout. Black lines indicate strong resting state functional connections between brain regions. The thickness of the lines indicates the relative connection strength (r). A spring-embedding algorithm (Net-Draw) was used to generate the 2D graph layout (Kamada and Kawai, 1989). This algorithm treats each connection as a spring; thus, brain regions with similar patterns of connections are brought closer together in 2D space. This method arranges the nodes of a graph in ‘connection space’ rather than anatomical space. Regions sharing connections are placed close together, whereas minimally connected regions are spatially distant. For example, the left and right IPS have similar connectivity profiles and are therefore positioned closely adjacent in the network graph. For each region (circle), the central color indicates which network it belongs to (black = cingulo-opercular; blue = cerebellar and yellow = frontoparietal). The outer color indicates the predominant control signal type of each region (red = set-maintenance; blue = error-related and yellow = start cue-related). At the displayed correlation threshold (r ≥ 0.15), the cingulo-opercular and frontoparietal networks are not directly connected to each other, but each network is connected to the cerebellar error-network through regions that also carry error information (the thalamus, dlPFC and IPL). This architecture suggests that both networks might be communicating error signals (or codes) to and from the cerebellum, in parallel. (b) Distinct cingulo-opercular (black) and frontoparietal (yellow) control networks, in addition to cerebellar regions (blue circles) are shown on an inflated surface rendering of the human brain. Caption and figure reproduced with permission (Dosenbach et al., 2008).

Network neuroscience research began around the turn of the century and has continued to develop to uncover the intrinsic functional structure of cognitive systems in the human brain. Cognitive functions like attention that can be studied with robust repertoires of precise tasks and stronger hypotheses about the systems involved can support more satisfying tests of possible mechanisms (Medaglia et al., 2015). Correlative studies have revealed that some networks recovered from intrinsic functional connectivity mapping at the group level spatially correspond with different networks associated with attention (Power et al., 2011). Graph theory paired with hierarchical clustering in intrinsic functional connectivity MRI data revealed that cognitive control regions separate into two intraconnected but distinct networks FPCN and CO networks (Dosenbach et al., 2008) (see Fig. 4). Interestingly and as previously discussed, these intrinsic networks have been implicated in various attention functions, to correspond to the cognitive control and salience demands, respectively, and it may be possible that their degree of functional connectivity is related to attention function overall (Seeley et al., 2007).

According to an early review of the psychological, functional anatomical, and cellular analyses of visual orienting, attentional processes have been functionally linked to oculomotor processes (Corbetta, 1998). Later, it was proposed that there are two partially distinct networks that are involved in controlling attention, with a dorsal frontoparietal network that is responsible for top-down processes and a ventral frontoparietal network that detects salient stimuli and acts as a ‘circuit breaker’ for the dorsal network (Corbetta and Shulman, 2002). Further supporting this notion within the same line of research, these dorsal and ventral systems were also found by correlating spontaneous fluctuations in brain activity at rest; furthermore, particular regions within the prefrontal cortex were found to interact with both systems, suggesting that these regions may be responsible for the interaction between the dorsal and ventral systems (Fox et al., 2006). Thus, most research thus far has found a distinction between the dorsal and ventral networks and described the functions that they are responsible for, but more recently, scientists have begun to question if these networks are solely responsible for the functions that they have been labeled with. For instance, it was suggested that the ventral system itself may play a general role in switching between the dorsal and ventral networks instead of being solely responsible for redirecting attention to behaviorally relevant stimuli (Corbetta et al., 2008). Potentially elaborating on this point, resting state functional connectivity analyses found that the temporoparietal junction (TPJ) and inferior frontal gyrus (IFG), both key components to the VAN, are strongly functionally connected to each other but are differentially activated (Shulman et al., 2009). The TPJ is activated during stimulus-driven reorienting while the IFG is only activated by unexpected shifts, suggesting that their roles are dissociable. Additionally, because the TPJ acts as a switching mechanism, this provides evidence that the VAN may be involved in switching between dorsal and ventral systems. Apart from these revelations for the VAN, the resting state functional connectivity of the DAN has been assessed and it has been found that during a visuospatial attention task, connectivity within the visual cortex is decreased, connectivity within the DAN is enhanced, and the visual cortex and DAN are more strongly functionally connected for the duration of active attention, suggesting a role for the DAN acting as a “prior” for attention selection (Spadone et al., 2015). The FPCN has been of great interest in network neuroscience research and particularly, it has become a key topic for functional connectivity studies. It has been found that the anterior cingulate, a region previously implicated in the FPCN, was activated during the presentation of an unexpected stimulus (Shulman et al., 2009). The cingulate has been argued to be a part of the executive control networks and is considered to control other networks to reflect current goals (Posner et al., 2007). Therefore, the FPCN is often described as an executive network involved in attention to deal with the problem of resolving conflict when selecting between responses (Posner, 2012). The FPCN has also been further divided into distinct subsystems responsible for different functions. The hierarchical clustering and machine learning classification analyses of within FPCN functional connectivity, revealed two subsystems of the FPCN that are functionally coupled with the DMN and the DAN, respectively (Dixon et al., 2018). One subsystem showed stronger connectivity with the DMN and is suggested to be involved in the regulation of introspective thought, while the other subsystem showed stronger connectivity with the DAN and is thought to be involved in the regulation of visuospatial perceptual attention.

### 4.2 The relationship between task activation and functional connectivity

There has been both activation and connectivity-based research that has each better informed our understanding of attention; however, the extent to which the same or different systems are engaged remains a question to be answered. From network neuroscience research using functional connectivity to predict activation during tasks, it is known that task activation for a set of tasks involving various rule complexity appears to be organized within functional networks (Cole et al., 2014); generally speaking, the brain's intrinsic network architecture at rest shapes the brain's functional network architecture during task performance. However, broadly speaking, it is unclear how cognitive functions are represented in the intrinsic organization of the brain, and whether fMRI appropriately represents this organization. In the case of attention, it is not clear that there are straightforward connections between task-evoked MRI networks and resting-state networks, and research on how each of these networks plays a role in basic attention processes as a whole is quite limited. Particularly, perspectives using traditional fMRI and connectivity reveal different spatial patterns of organization related to attention that have not been directly integrated. It is also unknown if the networks that activate in an attention task are the same networks that support those tasks due to the correlative nature of neuroimaging. Additionally, behavioral experiments can manipulate behavior and the associated changes in neural activity can be measured, but it is conceptually fraught to infer direct neural causality on this basis alone (Silvanto and Pascual-Leone, 2012). Therefore, it is important to have strong, causal manipulations to make inferences about the role of intrinsic and task-evoked networks in attention, and whether they represent similar or distinct systems.

### 4.3 Anatomical connectivity

Although functional imaging has been the primary focus of this review, it is imperative to mention how structural imaging and anatomical connectivity has played a role in revealing cognitive capabilities. For example, tractography imaging has revealed that anatomical variability, distribution, and volume of white matter fibers can predict the lateralization of visuospatial attention (De Schotten et al., 2011). Moreover, individual differences in the various white matter connections involved in visual attention processes are able to predict response to TMS (Cazzoli and Chechlacz, 2017; Chechlacz et al., 2015; Schintu et al., 2021). While we focus on the importance of clarifying the role of individualized functional networks, the joint contributions of anatomy and function will be an important frontier in neuromodulation research.

## 5 Transcranial magnetic stimulation (TMS) in the study of attention

**Fig. 5:**
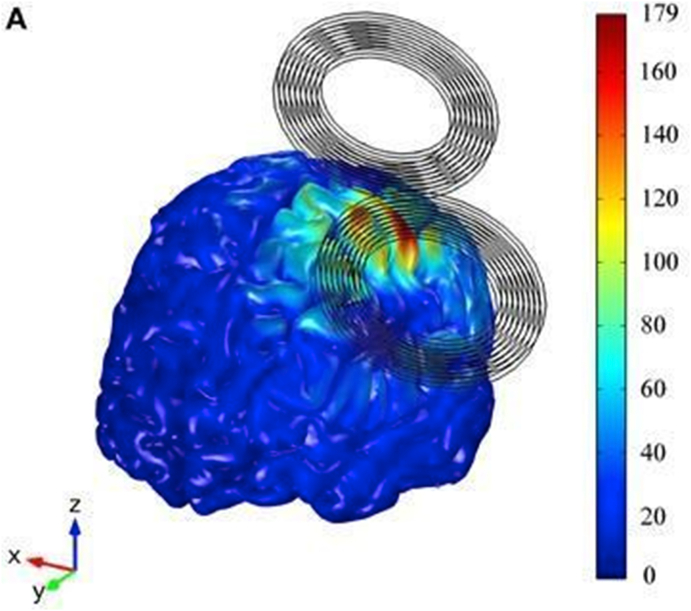
*Distribution of the E-field's magnitude induced during TMS*. The figure shows an E-field's magnitude in the GM volume using a common figure-eight style coil. Figure and caption reproduced with permission (Salvador et al., 2015).

It would be preferable to complement the neuroimaging of attention networks with direct neural stimulation to make stronger causal inferences. Our confidence in models of attention in the brain can be improved with careful experiments that manipulate attention-related behavior with well-defined tasks paired with direct neural stimulation and neuroimaging. Noninvasive brain stimulation paired with the appropriate behavioral tasks and neuroimaging could offer a strong multimodal tool to test for causality. Specifically, transcranial magnetic stimulation (TMS) could be an effective form of brain stimulation to influence these networks under varying cognitive demands because its peak influence can induce post-synaptic potentials and can be spatiotemporally precise compared to other noninvasive techniques (Hanlon, 2017). TMS is a form of non-invasive brain stimulation that uses electromagnetic induction to induce a short capacitor discharge of electric current into a coil which then generates a magnetic field; the magnetic field subsequently induces neuron potentials to change in cortical tissue under the coil (Zhengwu et al., 2018). The field's magnitude is strongest directly under the center of the coil and rapidly dissipates with further distance from center (Salvador et al., 2015) (see Fig. 5), and many common figure-eight coil designs support a spatiotemporal scale of about 1 cm³ with millisecond-scale discharges. Other ideas for how neuromodulation influences the cortex is via GABAergic neurotransmission (Trippe et al., 2009) and the expression of proteins and hormones in rat brains (Keck et al., 2000). Though, the most appeal for the use of TMS to researchers may simply be because it can depolarize or hyperpolarize neurons, causing action potentials. This direct excitation or inhibition of neural firing can help test the relationship between local neural activity and cognitive functions. Other common forms of neuromodulation, such as transcranial direct current stimulation (tDCS) for example, can also help to establish these relationships but because tDCS can only make a cell more or less likely to fire, TMS is the preferable form of noninvasive neuromodulation. Additionally, direct evidence for TMS influencing cortical reorganization and neural plasticity has been found. Applying TMS to induce a temporary lesion to a distinct network in one hemisphere can result in immediate compensation by the contralesional hemisphere to take over the lesioned networks functions (Sack et al., 2005). Furthermore, compensatory increases in the right premotor cortex and medial premotor areas have been observed, even without explicit behavioral effects following inhibitory TMS applied to the left premotor cortex (O'Shea et al., 2007). Finally, plasticity for aphasia recovery was observed in the right inferior frontal gyrus following inhibitory stimulation with TMS to the left homologous cortical area (Hartwigsen et al., 2013). Thus, TMS is useful because it has the capability to further explain how networks are organized, what their disparate functions may be, and how these networks can be plasticly altered following perturbations. With all of these uses and effects in mind, it can easily be seen that by targeting specific networks with TMS, a stronger causal link between neurons embedded within larger scale networks and behavior may be revealed.

There are several types of TMS that can be applied in research. The two most used forms are repetitive stimulation and theta-burst stimulation. Repetitive TMS (rTMS) involves sending repeating pulses of a particular frequency to the scalp and the stimulation effects are dependent on the frequency. Low frequency, typically 1 Hz, is considered to be inhibitory while 10 Hz and above is considered to be excitatory. Another common form of stimulation is theta-burst stimulation (TBS) which is a protocol that was derived from rTMS where researchers were able to send repeated application of bursts of pulses to the human motor cortex (Huang et al., 2005). TBS is thought to enact stimulation protocols similar to those seen in animal models to induce long term potentiation or depression (Huang et al., 2007). Therefore, TBS involves sending patterns of short bursts at high frequencies and can be given in continuous or intermittent protocols. Continuous theta-burst stimulation (cTBS) is believed to be an inhibitory protocol with stimulation effects reflecting a long-term depression-like decrease in neural activity. Intermittent theta-burst stimulation (iTBS) is believed to be an excitatory protocol with stimulation effects reflecting long-term potentiation-like increases in neural activity. These are the most conventional stimulation sequences applied in research and clinically, and their effects were validated in studies of the motor cortex using outcome measures like motor-evoked potentials (Di Lazzaro et al., 2004; Malcolm et al., 2006; Nitsche and Paulus, 2000). The application of these protocols to other areas of the human cortex thought to mediate cognition is our focus, and therefore it is crucial to discuss stimulation effects beyond the motor cortex. However, due to the lack of clear, temporally fast outcome measures in other cortical areas, it has been difficult to obtain valid and reliable markers of inhibitory and excitatory activity, leading investigators to principally use these sequences in research and clinical paradigms. Thus, there is a future need to obtain estimates of cortical and behavioral responses using these and other parameters. A more recent research avenue to reconcile this issue is the use of combined TMS-EEG to obtain potential markers of excitatory and inhibitory activity (Bortoletto et al., 2015; Du et al., 2018a, 2018b); this methodology will be further reviewed in a later section.

To date, the research aimed at modulating specific attention networks with TMS has been primarily centered around attention deficits following stroke, such as hemi-spatial neglect. There has not been a large selection of research in this arena that has aimed to ask basic science questions about attention network organization and function or that has involved other attention-based disorders or symptoms. However, a few studies have pointed to important questions about attention mechanisms and open questions that can be addressed with multimodal behavioral, imaging, and TMS designs. Here we describe TMS research with healthy and clinical populations to motivate our discussion of key questions for attention processing. Specifically, the clinical population of focus is patients with attention deficit/hyperactivity disorder (ADHD), whose disorder is almost purely centralized to deficits involving attention. In healthy subjects, two brain regions have been the principal locations of stimulation for research conducted for the effects of TMS on attention: the posterior parietal cortex (PPC) and the dorsolateral prefrontal cortex (DLPFC), while research involving subjects with ADHD has focused only on the DLPFC. Important to note, the PPC and DLFPC regions stimulated within the following studies used anatomical conventions to guide their targeting.

### 5.1 Healthy subjects

There have been three studies which have tested the effects of TMS on attention in healthy subjects, all of which stimulated brain regions revealed by the activation-based literature: Xu et al. (2013), He et al. (2013), and Xu et al. (2016). A commonality among these studies was that researchers measured attention with the Attention Network Test (ANT), which is a task that measures the efficiencies of the three putative sub-systems of attention (Fan et al., 2002). The ANT offers researchers the ability to separate the basic alerting, orienting, and executive inhibition components of attention. We chose to include *only* these studies because they include a TMS manipulation for relatively focal stimulation and an attention task that facilitates tests of these specific alerting, orienting, and executive components of attention in healthy subjects. The primary difference between the studies was the stimulation protocol used.

**Fig. 6:**
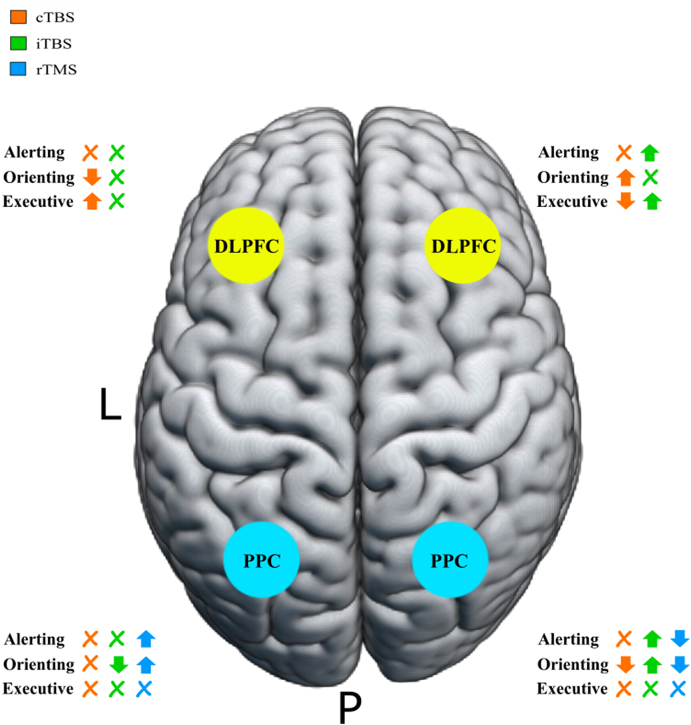
*A comprehensive figure depicting the effects of cTBS, iTBS, and rTMS on the three sub-components of attention: alerting, orienting, and executive control*. Xs indicate that there was no significant change in performance of the sub-function of attention as compared to the sham condition. An arrow pointing down indicates a deficit in performance for that sub-function. An arrow pointing up indicates an improvement in performance for that subfunction. Inhibitory stimulation to left hemispheric regions and excitatory stimulation to right hemispheric regions improve attention-related behaviors.

Xu et al. (2013) conducted a study applying cTBS to both the left and right PPC and DLPFC to study the role of different brain regions in visuospatial attention. Left DLPFC cTBS reduced performance for spatial orienting but increased performance for executive control, whereas right DLPFC cTBS increased performance for spatial orienting and decreased performance for conflict resolution. In contrast, left PPC cTBS yielded no significant changes in performance for alerting, orienting, and executive control. Right PPC cTBS reduced performance for spatial orienting. He et al. (2013) conducted a similar study, instead using iTBS to target the left and right PPC and DLPFC to create lasting increase in cortical excitability to investigate if hyperactivity of one hemisphere leads to hypoactivity in the contralateral hemisphere (i.e., a test of interhemispheric inhibition in the attention system). Left DLPFC iTBS yielded no significant changes in performance for any of the three functions of attention whereas right DLPFC iTBS increased performance for alerting and executive control. In contrast, left PPC iTBS reduced performance for spatial orienting. Right PPC iTBS increased performance for both alerting and spatial orienting. Finally, Xu et al. (2016) conducted a study applying low frequency inhibitory rTMS to the left and right PPC to potentially identify a brain network responsible for spatial cognition. Left PPC rTMS increased alerting and orienting performance, while right PPC rTMS decreased performance. The findings from the aforementioned studies have been summarized in Fig. 6.

Specifically, these results reveal a thematic relationship between brain region, type of TMS administered, and subsequent behavior. Applying cTBS (inhibitory) to the left and right DLPFC revealed a difference in executive control performance, causing increased performance in the left DLPFC and worsened performance in the right DLPFC, suggesting that the right DLPFC plays a central role in executive control behaviors that might be enhanced via inhibiting its left hemispheric homotope. cTBS to the left and right PPC revealed no influence over executive control performance, suggesting that the DLPFC's specific role in executive control remains stable when posterior regions that interact with the DLPFC during executive control are stimulated. However, TMS experiments suggest that the PPC especially in the right hemisphere is involved in orienting behavior, as performance worsened because of inhibitory stimulation. Therefore, there is a trend that inhibiting attention-related regions in the left hemisphere or exciting them in the right hemisphere may improve performance.

### 5.2 ADHD subjects

There are only two studies that will be reviewed in this paper that have observed the effects of using TMS to treat attention deficits in ADHD patients. However, it is important to note that these are not the *only* studies that have used TMS to study attention in clinical subjects. As previously noted, there has been a handful of research using TMS in stroke patients to study and treat attentional neglect (Fierro et al., 2006; Muggleton et al., 2006). However, while these studies are crucial in better understanding stimulation effects on cognition, such neglect studies will not be included in this review because these studies involve patients who have lesioned brains resulting from stroke or brain injury. Instead, we will focus on studies involving patients with anatomically intact brains, particularly, those diagnosed with attention deficit hyperactivity disorder (ADHD): Bloch et al. (2010) and Weaver et al. (2012). By only reviewing studies that have aimed to treat the attention deficits in ADHD subjects, we can more directly focus on how researchers have attempted to utilize non-invasive brain stimulation to treat the major tenets of attention, such as top-down processing. However, it is important to point out that unlike the studies involving TMS and healthy subjects, these studies utilized solely subjective measures to evaluate attention performance. While subjective measures are important for gaining first-hand experience information from the patient, well-defined, reliable, and robust tasks that objectively measure attention performance will be necessary for accurately measuring the stimulation effects on attention behavior. Additionally, this should provide support for the notion that more research with clinical subjects, like those with ADHD, should be conducted that utilize objective cognitive and behavioral tasks. Nonetheless, despite the lack of explicit behavioral tasks to measure attention, these studies are pertinent to this review, as they are the major studies that have used TMS to treat attention deficits. In these studies, subjective behavioral measures were given before and after excitatory rTMS applied to the right DLPFC. Bloch et al. (2010) applied excitatory 20 Hz rTMS to the right DLPFC in 13 adults with ADHD. The Positive and Negative Affect Schedule (PANAS) questionnaire was given as a self-report measure to evaluate several emotions, including attention, hyperactivity, anxiety, and mood; attention and hyperactivity scores were combined to create an overall ADHD score. Visual analog scales (VASs) for attention and mood were also given as self-report measures to evaluate current affect states. Right DLPFC rTMS was found to significantly improve the overall ADHD score, while no significant changes were seen for anxiety or mood. VAS scores for attention also improved following stimulation.

Weaver et al. (2012) applied excitatory 10 Hz rTMS to the right prefrontal cortex in 9 adolescents and adults with ADHD. The study included a randomized, sham-controlled, crossover design in which participants completed 2 weeks of either sham or stimulation treatment, followed by 1 week of no stimulation, followed by 2 weeks of the other treatment type that they had not been assigned to in the first 2 weeks. The Clinical Global Impression – Improvement Scale (CGI-I) was used in this study and psychiatrists assessed the participants’ ADHD symptoms throughout the duration of the study. The ADHD-IV scale was also given as a secondary measure to evaluate ADHD symptoms. Right DLPFC rTMS was found to improve CGI-I scores; however, this improvement was seen in sham as well as active stimulation groups within the first 2-week phase of stimulation. However, only active stimulation improved scores over the second 2-week phase. This suggests that there may be placebo-like effects early in treatment, but once these effects plateau, subjects might benefit more from active stimulation relative to sham. Moreover, there were trends toward significant changes in both the CGI-I and the ADHD-IV scales for the active TMS group more than in the sham group, but these differences between conditions were not significant. The authors suggested that TMS may have positive clinical applications for ADHD patients that would be worth studying in larger samples.

Additionally, the results of these studies provide evidence in clinical samples that excitatory stimulation to the right DLPFC improves ADHD symptoms, perhaps as related to executive control behaviors. Encouragingly, these findings are consistent with studies in healthy subjects suggesting that right-hemispheric excitation or left-hemispheric inhibition can improve executive attention performance. As noted, the addition of precise behavioral paradigms that measure attention over subjective behavioral measures would provide more direct evidence for the stimulation effects on attention in these ADHD patients. In addition, as previously mentioned, the reviewed TMS studies here used anatomical conventions to define their targets for PPC and DLPFC stimulation. Using such methods creates ambiguity and distinct limitations within this line of research, as researchers cannot directly determine which neural networks are being engaged with PPC or DLPFC stimulation.

### 5.3 Limitations to prior TMS research

It is important to note that although the previously discussed studies are the first of their kind to stimulate attention networks with TMS to observe behavioral changes, there is reason to believe that the targeting methods used are not optimal to achieve the desired outcomes. Briefly, within the healthy participant TMS research, the DLPFC and PPC stimulation points were chosen via a 10/20 EEG placement system: the DLPFC target was chosen as left or right F3/F4 labels and the PPC target was chosen as the left or right P3/P4 labels. Within the clinical participant research, the DLPFC target was chosen by measuring 5 cm anterior from the motor threshold location. This methodology is based on early literature on the use of TMS as a treatment for depression, and the “5 cm rule” and “5 + 1 cm rule” have since been a standard for stimulating the prefrontal cortex (George et al., 1995, 1996; Pascual-Leone et al., 1996). These methods are concerning because they do not reliably identify either underlying neuroanatomy or functional circuits. For example, the 10–20 EEG system does not consistently identify the brain regions that an electrode lies over. Electrodes F3 and F4 have been attributed to the middle frontal gyrus, specifically Brodmann's areas 46 and 9 (Homan et al., 1987), but have also been attributed to being above Brodmann's areas 8 and 9 (Herwig et al., 2003). In addition, it has been shown that using the “5 cm rule” identifies different sites for stimulation across different people (Ahdab et al., 2010). When aiming to target the DLPFC using this method, it has been shown that locations above the premotor cortex have been unintentionally targeted (Herwig et al., 2001).

Further complicating the neurotargeting literature is the fact that attention networks (like many networks) might be represented differently across subjects. Neuroimaging evidence reveals that the location of a functional network hub is highly variable across people and is only partially constrained or predictable through anatomy (Feredoes et al., 2007). In addition, attention endophenotypes might be different in various people with different neural bases. Therefore, targeting individual subjects using a group-level average targeting method, such as an EEG cap or the “5 cm rule,” is far from ideal. By using these targeting methods, the probability of stimulating a single network in every individual's brain is unlikely when using an average target. As a result, it can be assumed that prior studies stimulated many different networks across persons and not just those of specific interest.

Therefore, even within the TMS literature, some questions still remain regarding the neural causes of behavior. Do we know if we are inhibiting or exciting the exact network hub of interest that is responsible for the behavior with the stimulation, and how can we tell if we were? Does an inhibitory or excitatory effect reveal anything about what is happening in the neural circuits? Moreover, could understanding subject-specific representations of attention networks explain TMS behavioral effects, elucidate brain network-behavior relationships, and point to translational benefits?

To address these questions, we suggest that TMS research should focus on finding *person-specific targets* for stimulation. This could allow us to enhance the power of basic studies (Sack et al., 2009), and refine targets for clinical use. By combining computerized tasks, noninvasive brain stimulation (e.g., TMS), and personalized intrinsic network mapping using fMRI, we could provide stronger tests of the basis of attention, reconcile traditional fMRI and connectivity studies, and discriminate among theories of network function. Moreover, they could provide a mechanism-based, specific means to develop treatments for existing dysfunction than existing pharmaceutical and behavioral approaches.

## 6 Toward understanding specific attention functions in single subjects

**Fig. 7:**
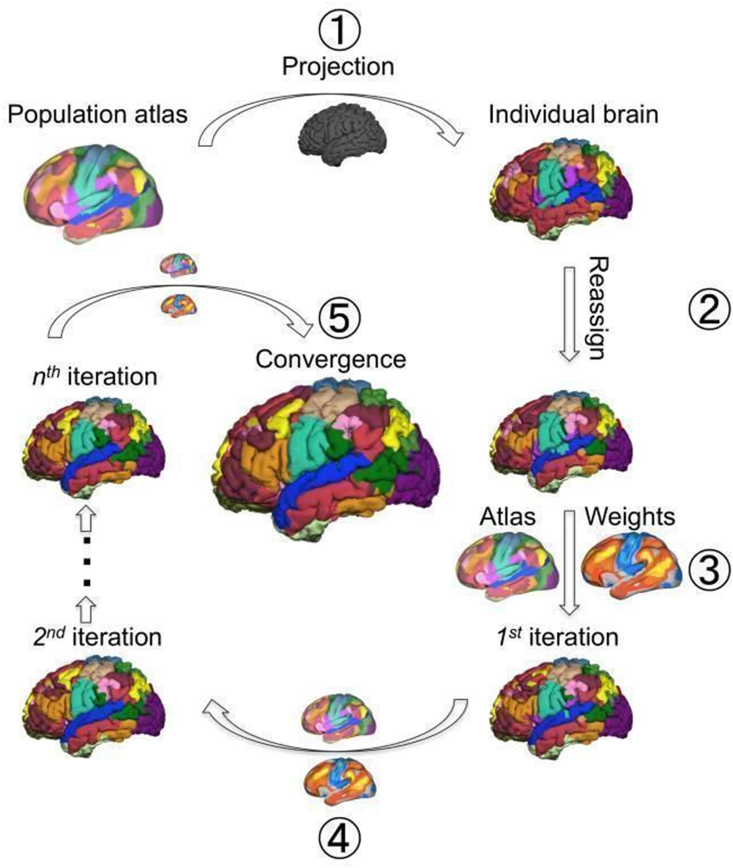
*Parcellating the functional networks in an individual subject's brain using an iterative adjusting approach.* The technique includes the following steps: 1) A population-based functional brain atlas was registered onto the individual subject's cortical surface using FreeSurfer. The individual subject's BOLD signal time courses were then averaged across the vertices that fall within each network. These atlas-based network time courses were used as the “reference signals” for the subsequent optimization procedure. 2) The individual subject's BOLD signal at each vertex was then correlated to the 18 “reference signals”. Each vertex was reassigned to one of the 18 networks according to its maximal correlation to the “reference signals”. A confidence value was also computed as the ratio between the largest and the second largest correlation values. After each vertex was reassigned, the BOLD signals of the high confidence vertices (e.g., >1.1) in each network were then averaged and termed the “core signal”. 3) For each network, the “core signal” derived from Step 2 and the original “reference signals” derived from Step 1 were averaged in a weighted manner. Specifically, the “core signal” was multiplied by the weighting parameters derived from inter-subject variability and SNR, as well as the number of iterations. The averaged signal was used as the new “reference signal” for the next iteration. Using these new “reference signals”, the brain vertices were further reassigned to one of the 18 networks. 4) Steps 2 & 3 were repeated until the algorithm reached a pre-defined stopping criterion. Figure and caption adapted with permission (Wang et al., 2015).

Fortunately, new methods are becoming available to support single-subject network mapping for exactly these ideas. Work in within-subjects precision network mapping has shown that person-specific targets can be derived from resting-state fMRI parcellations of the cortical surface from a single scanning session (Gordon et al., 2017; Wang et al., 2015) (see Fig. 7), which could improve the specificity of stimulation effects. These parcellations could be used in traditional test, stimulate, and test paradigms (e.g., before and after the subject enters the scanner), or simultaneously with fMRI. For instance, to test the mechanism of action of TMS application to the dorsolateral prefrontal cortex in major depressive disorder treatment, researchers used TMS-fMRI to reveal the propagation of induced neuronal activity associated with stimulation (Vink et al., 2018). Concurrent TMS and fMRI is therefore useful to map neural circuits and uncover how brain regions communicate with one another, given its ability to measure spatially precise cortical and subcortical changes in activity following perturbation via stimulation (Bergmann et al., 2021; Oathes et al., 2021).

**Fig. 8:**
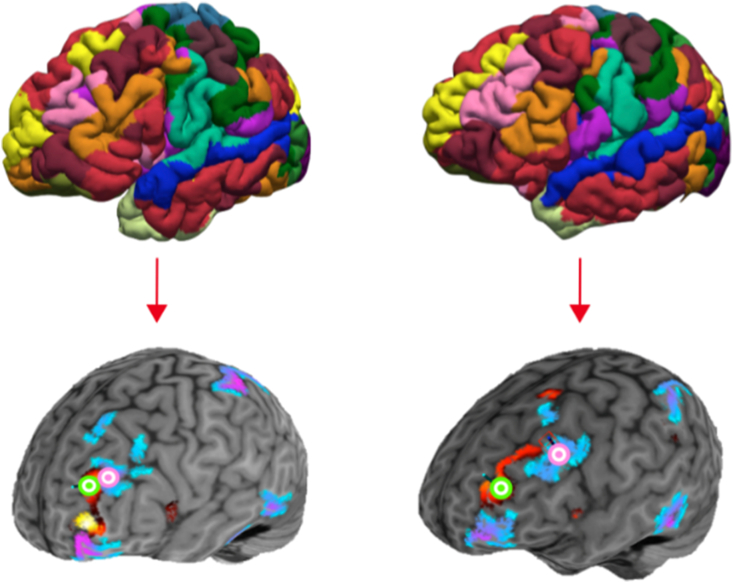
*Two brains run through a parcellation method to create individualized network maps.* The various colors in the top-most photos represent the same networks in two different subjects. However, their expression is clearly not the same. The bottom-most photos display two distinct brain networks between brains. The lime green bullseye refers to a target for stimulation of the red network. The pink bullseye refers to a target for stimulation of the blue network. It is clear that these networks and their targets are not in the same location between subjects which corroborates the idea that group-level targeting is not optimal nor accurate.

An attractive feature of the specific parcellation method presented in Fig. 7 is that this method has been validated with invasive cortical stimulation, further supporting its use in creating individualized network maps that can estimate intrinsic networks within each subject and allow for more precise targeting. Fig. 8 shows two different brains from our own data which have been parcellated with the same method. It is clear from these parcellations that the estimated brain networks are not exactly similar between brains, and do not respect identical anatomical boundaries, which further suggests that group-level average targeting for any particular network is not optimal. For our current purposes, it is important to note that it is not known if the network hubs themselves (Lynch et al., 2019) or the connections between networks are most relevant to attention performance. To test this, precision targeting methods using individualized network parcellations should be used in order to find the exact location for stimulation. The network should be selected based on a hypothesis about its role in cognitive function (here, attention). Then, TMS may be used to target these specific network hubs or the connections between them to better understand their precise role in attention by observing the degree to which each modulates its mean activity, connectivity, and behavior. This methodology may help improve TMS effects for modulating attention function by increasing stimulation location specificity. It may also reveal a differentiation between effects from stimulating network hubs and network connections for attention performance. Possibly, it could be that network connectivity between brain regions previously shown to activate in attention tasks is more responsible for performance than network connectivity within those networks. Although, there may be an opposite result, revealing that within network connectivity of those regions that subserve different attention functions is more important for performance and does not rely on between network connectivity. Furthermore, the degree of functional connectivity between and within brain regions may play no causal role in attention performance and thereby suggest that activation-based literature provides an adequate representation of attention in the brain. However, from the research reviewed here revealing the co-activation of brain regions involved in each of the attention functions, it is possible the degree of network connectivity *between* these regions will be more relevant to performance than within network connectivity and activation alone.

It should also be mentioned that there are multiple ways to define group and individualized parcellations, with investigators using network clustering techniques or independent component analysis: (c.f., Salehi, Karbasi, Scheinost, & Constable (Salehi et al., 2017); Salehi, Karbasi, Shen, Scheinost, & Constable (Salehi et al., 2018); Salehi, Karbasi, Barron, Scheinost, & Constable (Salehi et al., 2020b); Han et al. (2020); and Chong et al. (2017)). Validating such parcellations and their role in cognitive functions with multiple methods including TMS or other neural stimulation and recording techniques in individual subjects is an important direction for future research. Furthermore, the reliability and validity of these methods in clinical samples must be evaluated, and the predictive validity to clinical status and TMS outcomes should be tested.

## 7 Complementary methods – TMS, EEG, and fMRI

While we have emphasized using personalized fMRI mapping, it is important to remark upon other anatomical and functional methods that could enhance single-subject neuromodulation.

In addition to TMS-fMRI integrated studies, TMS has been combined with EEG and has been important in uncovering temporally precise information regarding the human connectome. Specifically, integrating these techniques may allow researchers to better understand the brain rhythms involved in cognition and behavior through direct manipulation of cortical frequencies with TMS and temporally precise recordings with EEG (Thut and Miniussi, 2009). TMS-EEG has also been helpful in assessing neural networks, revealing information about cortical excitability, effective connectivity, and oscillatory tuning of different brain areas (Rogasch and Fitzgerald, 2013). This set of methods also has great clinical utility in identifying pathophysiological biomarkers for psychiatric and neurological disorders and in predicting treatment response (Tremblay et al., 2019). Recently, investigators have achieved closed-loop protocols with TMS and EEG, where TMS pulses are triggered by a given EEG signal which may improve stimulation effects (Moreno et al., 2021; Shirinpour et al., 2020).

Combining TMS, EEG, and fMRI has also been implemented to identify state-dependent effects and cause time-dependent activation. This multimodal application in research has allowed for direct neural manipulations while measuring spatially precise brain activity in response as well as measuring temporally precise fluctuations in cortical oscillations (George et al., 2019; Peters et al., 2020).

## 8 The challenge of behavioral variability in attention

Prior behavioral studies on attention have used the ANT to measure subjects' behavioral efficiency of several attention subfunctions. Efficiency scores on the test are calculated to measure the brain's alerting, orienting and executive system as a function of reaction time (RT) and error rate (Ishigami and Klein, 2010). However, RT and error rate are both typically measured as a mean function across people on average, as opposed to being measured over time within subjects. Thus, the focus of this research has been across people on average and not how attention evolves within specific persons and networks over time. Additionally, researchers will measure reliability as a function of correlating individual mean RT and mean error rate only for the first two sessions or blocks of trials (Ishigami and Klein, 2010). Mean RT scores of the ANT are often tested using an Analysis of Variance (ANOVA) using the session as the repeated measures factor to understand performance across sessions (Ishigami and Klein, 2011). This study design was carried out to understand differences in reliability of two versions of the ANT (ANT and ANT-1), and it revealed that a lack of independence among the three putative networks was not independent for both versions. Additionally, reliability began to decrease as more sessions were included in the statistical analysis for each individual. It is difficult to assert that the ANT is highly reliable considering the lack of conclusive and consistent studies on this subject (Hahn et al., 2011).

However, low reliability must be distinguished from meaningful response variability. While the statistical reporting mean ANT RT correlations has been consistent across studies, the use of averages across individuals eliminates quantifiable individual differences across sessions, as well as the ability to relate intra-subject variation to individual differences in networks. Additionally, many researchers utilize split half reliability correlations, which eliminates the ability to see changes in each network for each individual, as it typically involves comparing scores from the first two blocks to the last blocks (MacLeod et al., 2010). For instance, data from 15 studies with a total of 1129 healthy individuals calculated the split-half reliabilities of RT attention network scores; the results indicated that the networks that the three ANT efficiency measures are not completely independent. Additionally, ANOVAs indicated that the power to find any detectable significant effect was variable across the networks and, also, that the type of statistical analysis being utilized had an effect (MacLeod et al., 2010). The reliability of a measure is important because the potential low reliability of a measurement, such as the ANT, can increase the potential of finding statistically significant differences in attention. There are two problems that need to be addressed: how attention is represented in a single person's brain over space and time, and how this knowledge could enhance clinical progress in the study of attention disorders.

## 9 Integrating methods for precision modulation and cognitive discovery

**Fig. 9:**
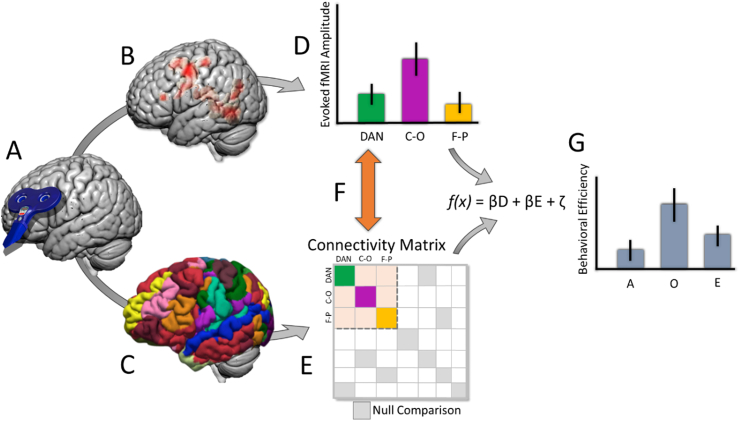
*Integrating methods for precision modulation and cognitive discovery.* (A) Neuromodulation can be applied to modulate brain activity by stimulating brain networks (B) Brain stimulation can increase or decrease brain activity in specific regions. (C) The brain can be parcellated into distinct neural networks to reveal an individual's network organization. This parcellation can show where specific networks are within subjects. (D) Evoked amplitude can be measured across specific brain networks, here specifically the dorsal attention network (DAN), cingulo-opercular network (C–O), and frontoparietal network (F–P), to understand how networks activate during a task. (E) Functional connectivity of networks can be measured to understand how networks temporally activate during a task or at rest. This connectivity matrix reveals how strongly a network is connected to itself or other networks (e.g., as the sum of connectivity weights such as correlation or coherence within and between networks). Here, the functional connectivity of the same networks described in (D) could be more strongly predictive of behavior and activation than other networks. This hypothesis can be tested with models trained using data from equally sized network connectivity values from other networks. (F) Connectivity could predict functional activation within networks. (G) Attention-related behavior can be predicted based on the (D) activation-based research or the (E) connectivity-based research as a combined weighting of the activation and connectivity data. The bars representing behavioral efficiency are in grey because it is not known in advance whether one single network is responsible for one sub-function of attention; instead, it may be a combination of networks that impact behavioral performance. This defines the frontier of discovery for TMS-fMRI paradigms for attention.

To address the above-mentioned problems, we suggest integrating the methods described earlier for precision neuromodulation to better understand attention in the brain at the individual level (see Fig. 8). As mentioned previously, attention has been studied with an activation-based perspective and a connectivity-based perspective. Activation-based research has revealed how task-evoked amplitudes for specific networks involved in attention differ when completing a task. These amplitudes have been correlated to specific attention behavior. Furthermore, network connectivity has revealed how these attention networks are functionally connected to themselves and other networks; as well as activation, connectivity has also been shown to be correlated to attention behavior. However, it is unknown how activation and connectivity together predict behavior. Perhaps, activation within and between specific systems predicts the variance in attention behavior more than connectivity, or vice versa. Alternatively, it is possible that activation and connectivity provide additive predictive validity. Furthermore, although behavior can be predicted from both activation and connectivity, how each specific attention network relates to a specific subfunction – or set of subfunctions - of attention is not known. Because neuroimaging data is only correlative, a causal tool needs to be used to probe networks to understand their role in attention, such as TMS. In order to be sure that exact attention networks will be stimulated with TMS, precision targeting methods need to be employed. Using individualized network parcellation maps, an individual's specific attention network can be found and stimulated with neuromodulation. This precision targeting methodology can ensure that an exact network is being stimulated, and this may increase the effects of brain stimulation. By being sure that the exact network of interest is being stimulated, a more thorough understanding of that network's role in behavior, including its variability within subjects over time, can be revealed (see Fig. 9). Similar integrative methods have been suggested previously, such as combining lesion analyses and the human connectome to create lesion network mapping to improve treatment targets (Fox, 2018), which indicates that multimodal approaches can improve research and research outcomes in various disciplines. Crucially, there has been some recent empirical evidence for an integrative model similar to what we are suggesting for cognitive discovery. It has been shown through the combination of fMRI guided, individualized TMS, simultaneous EEG, and reliable empirical paradigms that discrete neural networks can be perturbed and measured, and that those measurements can act as biomarkers for cognitive performance (Ozdemir et al., 2020). The results from this study can provide us with necessary information about the organization and role of specific neural networks in cognitive and behavior, which can then later better inform our treatment plans and patient outcomes.

## 10 Conclusion

Attention is a complicated cognitive mechanism which has been studied with various methods and theoretical frameworks. It is imperative to better understand how attention processes are organized and carried out in the brain, and brain stimulation may be an effective tool to uncover this information. The past literature about the effects of TMS on attention has positive prospects in promoting our understanding of attention circuits and guiding interventions. Although more TMS research needs to be conducted to answer basic questions about attention circuits, researchers should meanwhile study multiple different clinical populations. The five studies reviewed here show evidence that TMS can modulate attention behavior with some parity between stimulation sequences and brain regions. Therefore, we may be able to utilize TMS to stimulate brain networks associated with attention to change behavioral outcomes in clinical populations. However, more research can personalize TMS parameters, such as targeting, to ensure that the strongest behavioral effects are being elicited within persons that provide the most stringent tests of the roles of specific networks. By improving targeting strategies, perhaps the variability of TMS effects will become lower and results will be more consistent. Once precise targets are found, we may be able to stimulate exact network hubs and their connections to improve our understanding of functional connectivity for attention and the mechanism overall. Finally, these impending results may provide positive implications for clinical applications of TMS by increasing stimulation effect specificity.

## Author contributions

**B.E. Yeager:** Conceptualization, Writing – original draft, Writing – review & editing. **C.C. Dougher:** Writing – original draft. **R.H. Cook:** Writing – review & editing. **J.D. Medaglia:** Conceptualization, Writing – original draft, Writing – review & editing.

## Declaration of competing interest

The authors declare that they have no known competing financial interests or personal relationships that could have appeared to influence the work reported in this paper.
