# Supplementary material for: The role of transcranial magnetic stimulation in understanding attention-related networks in single subjects
Source: Curr Res Neurobiol. 2021 Jul 3;2:100017. doi: 10.1016/j.crneur.2021.100017 (PMC9559099; doi:10.1016/j.crneur.2021.100017)
Supplement: Multimedia component 1 [file mmc1.pdf]

Peer Review Overview

Manuscript Title: The Role of Transcranial Magnetic Stimulation in Understanding Attention-Related Networks in Single Subjects

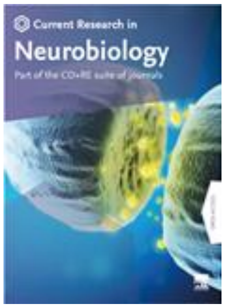

Received	Nov 18, 2020
1st Decision	Mar 11, 2021
1st Revision Submitted	May 24, 2021
2nd Decision	Jun 20, 2021
2nd Revision Submitted	Jun 22, 2021
Accepted	Jun 30, 2021

1st Decision letter

Reference: CRNEUR-D-20-00014
Title: The Role of Transcranial Magnetic Stimulation in Understanding Attention-Related Networks in Single Subjects
Journal: Current Research in Neurobiology

Dear Ms Yeager,

Thank you for submitting your manuscript to Current Research in Neurobiology.

I have completed my evaluation of your manuscript. The reviewers recommend reconsideration of your manuscript following major revision. I invite you to resubmit your manuscript after addressing the comments below. Please resubmit your revised manuscript by May 10, 2021.

When revising your manuscript, please consider all issues mentioned in the reviewers' comments carefully: please outline every change made in response to their comments and provide suitable rebuttals for any comments not addressed. Please note that your revised submission may need to be re-reviewed.

In addition, you might be interested in reading our Editorial Introduction, https://doi.org/10.1016/j.crneur.2021.100005 to the journal that was just published, and we would love to get your advice via the short survey, https://www.surveymonkey.co.uk/r/ZLN8GYL .

Current Research in Neurobiology values your contribution and I look forward to receiving your revised manuscript.

Kind regards,

Anna S Mitchell, Ph.D.
Editor in Chief
Current Research in Neurobiology

Comments from Editors and Reviewers:

Thank you for submitting your manuscript. Two experts in the field and myself have reviewed your work. You will see from the reviewer's comments that they both recommend including additional research papers to support your reviewing request that you consider condensing the initial historical section.
Please also ensure the references are accurate eg. #134 is Kastner and Ungerleider 2000.

Reviewer #1:

The Authors propose a review and position paper on the role of transcranial magnetic stimulation (TMS) in understanding attention-related networks in single subjects. They first review research concerning attention in an historical perspective, then present major themes in attention neuroscience, investigated brain networks, studies in non-human primates, neuroimaging research in stroke patients and in healthy individuals, and then come to TMS research. They propose that combing TMS with network analyses at the single-subject level can potentially help to better elucidate the neural underpinnings of attention in the human brain and even improve treatment approaches for attention disturbances. The manuscript is generally easy to follow and highlights different interesting aspects in current research on attention. However, I have several points that the Authors may want to consider:

* In general, considering the title (and the topic) of the manuscript, the part concerning TMS in the study of attention is proportionally quite small in comparison to the previous "introductory" sections.
* On a somewhat related note, bold statements such as "the past literature about the effects of TMS on attention is not extensive" (4th line of the Conclusions) or "there have been three studies which have tested the effects of TMS on attention in healthy subjects" (lines 1-2 of the section "Healthy subjects") do not seem to reflect the reality. There are several thousands of published studies investigating attention by means of TMS. It is obviously impossible (and beyond the scope of the review) to cover the whole literature. However, the Authors could probably tune down these statements and better justify why they select only certain studies (and why this small amount with respect to the scope of the review).
* The presentation of the "most commonly used forms" of TMS in research (2nd page of the section "Transcranial Magnetic Stimulation (TMS) in the Study of Attention") is not clear. For instance, "classic" repetitive TMS (rTMS) protocols do not entail bursts of stimuli, but "homogeneous" stimulation frequencies (as then mentioned in the following). Moreover, cTBS and iTBS are particular forms of rTMS (on a side note, references to work of the group that developed cTBS and iTBS are missing).
* Similar approaches as the one proposed in the review have already been postulated (e.g., Fox, 2018. Mapping symptoms to brain networks with the human connectome. New England Journal of Medicine, 379(23)) or applied (e.g., Ozdemir et al., 2020. Individualized perturbation of the human connectome reveals reproducible biomarkers of network dynamics relevant to cognition. Proceedings of the National Academy of Sciences, 117(14)). The authors may want to follow up on this literature.
* A perturbation of a given network node by means of TMS has been shown to elicit compensatory processes in other distant nodes (see, e.g.: Sack AT et al., 2005. The dynamics of interhemispheric

compensatory processes in mental imagery. Science 308; O'Shea J et al., 2007. Functionally specific reorganization in human premotor cortex. Neuron 54; Hartwigsen et al., 2013. Perturbation of the left inferior frontal gyrus triggers adaptive plasticity in the right homologous area during speech production. Proc Natl Acad Sci U S A 110). The Authors may want to follow up on this aspect.
* The review focuses on results stemming from fMRI research. However, several studies combing TMS and EEG have also shown to be able to deliver important information on the human connectome with high temporal resolution (see, e.g., for a review: Bortoletto et al., 2015. The contribution of TMS-EEG coregistration in the exploration of the human cortical connectome. Neurosci Biobehav Rev, 49). The Authors may want to integrate this perspective and/or comment on why to focus their perspective only on fMRI.
* The Authors consider virtually only functional connectivity. However, already seminal papers have shown that also structural connectivity (in terms of properties of white matters tracts connecting attention networks nodes) plays a central role in individual variations in attentional performance (e.g., Thiebaut de Schotten et al., 2011. A lateralized brain network for visuospatial attention. Nat Neurosci 14(10)). Moreover, these individual differences in structural connectivity also modulate the effects of TMS on attentional performance (e.g., Chechlacz et al., 2015. Structural Organization of the Corpus Callosum Predicts Attentional Shifts after Continuous Theta Burst Stimulation. J Neurosci 35(46); Cazzoli et al., 2017. A matter of hand: Causal links between hand dominance, structural organization of fronto-parietal attention networks, and variability in behavioural responses to transcranial magnetic stimulation. Cortex 86; Schintu et al., 2020. Callosal anisotropy predicts attentional network changes after parietal inhibitory stimulation. Neuroimage 226). The Authors may want to integrate this perspective in their review.
* It seems somehow odd to report only on ADHD as a model for the application of TMS in attention disorders. For instance, the Authors previously describe several empirical paradigms aimed at specifically assessing discrete aspects of attention in healthy individuals, but for the clinical application they report on three ADHD studies in which TMS effects on attention were assessed by means of subjective measures such as self-report and questionnaires.

Reviewer #2:

In the submitted manuscript the authors present an in-depth review of the neuroscience of attention in the context of extensive theoretical and empirical work, with a special emphasis on network neuroscience and non-invasive brain stimulation. Overall, this is an excellent review of the literature, and has the basis of many future empirical investigations. I only have a few conceptual issues that I would like the authors to address.
1. The first 5 pages provide a nice review of the current state of knowledge. However, I feel this is a bit distracting from the purpose of the review, which in my opinion, starts at Brain Networks and Attention. I am sure there are several other review articles and book chapters that have summarized historical, theoretical, and empirical perspective of attention, and perhaps these sections can be summarized more concisely in order to get to the main substance of the review article.
2. The author detail the conventional methods of applying TMS (iTBS, cTBS, 10hz rTMS and 1hz-rTMS). However, the excitatory/inhibitory effects of the protocol are largely based on motor cortex studies, particularly TBS. I think the author should discuss the stimulation effects of these protocol beyond the motor cortex, but more of the observed effects of these protocol on other brain regions, which sometime are incompatible with the dogma of these motor cortex studies.
3. In regards to conventional TMS methods, other methods have also been employed to study attention (closed-loop TMS, combined EEG - TMS). Animal TMS studies may also provide additional information

about the effects of TMS on attentional networks.
4. There has been a few recent studies using simultaneous TMS and fMRI to study brain networks (resting state). Perhaps adding these to the review is relevant here.
5. In regards to network science, the author should also review the implications of different methodological approaches to identify brain networks and quantify their correlations in the context of brain networks and mental disorders

1st Author Response Letter

Response to comments from Editors and Reviewers:

Comments from Reviewer 1

The Authors propose a review and position paper on the role of transcranial magnetic stimulation (TMS) in understanding attention-related networks in single subjects. They first review research concerning attention in an historical perspective, then present major themes in attention neuroscience, investigate brain networks, studies in non-human primates, neuroimaging research in stroke patients and in healthy individuals, and then come to TMS research. They propose that combining TMS with network analyses at the single-subject level can potentially help to better elucidate the neural underpinnings of attention in the human brain and even improve treatment approaches for attention disturbances. The manuscript is generally easy to follow and highlights different interesting aspects in current research on attention. However, I have several points that the Authors may want to consider:

● We thank the reviewer for many helpful comments. We hope that our responses and edits have improved the clarity and rigor of this manuscript and would be happy to integrate any further suggestions.

1. In general, considering the title (and the topic) of the manuscript, the part concerning TMS in the study of attention is proportionally quite small in comparison to the previous "introductory" sections.
● We agree that the proportion of the paper spent reviewing background information on attention deterred readers from our main points. Thus, we condensed the first 5 pages of introductory/background information while leaving main points, seminal literature, and defining key terms. We hope that our main focus comes through stronger and more clearly following these changes. History now being pages 2-5.

2. On a somewhat related note, bold statements such as "the past literature about the effects of TMS on attention is not extensive" (4th line of the Conclusions) or "there have been three studies which have tested the effects of TMS on attention in healthy subjects" (lines 1-2 of the section "Healthy subjects") do not seem to reflect the reality. There are several thousands of published studies investigating attention by means of TMS. It is obviously impossible (and beyond the scope of the review) to cover the whole literature. However, the Authors could probably tune down these statements and better justify why they select only certain studies (and why this small amount with respect to the scope of the review).
● We agree. We do not want to suggest the idea that there is hardly any research regarding the effects of TMS on attention; our language was audacious and we removed such statements. "The past

literature about the effects of TMS on attention is not extensive" from the Conclusion section on page 36 was changed to “The past literature about the effects of TMS on attention has positive prospects in promoting our understanding of attention circuits and guiding interventions. Although more TMS research needs to be conducted to answer basic questions about attention circuits, researchers should meanwhile study multiple different clinical populations.” We think this change better reflects this paper’s purpose, that there simply needs to be more research conducted with TMS and other integrative methods which could then allow us to come to a better understanding of attention. We also did want to address that a better understanding of attention will also come from studying multiple different clinical populations with TMS which the previous literature has not addressed.

● To elaborate on why we chose 3 TMS studies, on page 24 we write, “There have been three studies which have tested the effects of TMS on attention in healthy subjects, all of which stimulated brain regions revealed by the activation-based literature: Xu et al. [163], He et al. [61], and Xu et al. [164]. A commonality among these studies was that researchers measured attention with the Attention Network Test (ANT), which is a task that measures the efficiencies of the three putative sub-systems of attention [42]. The ANT offers researchers the ability to separate the basic alerting, orienting, and executive inhibition components of attention. We chose to include only these studies because they include a TMS manipulation for relatively focal stimulation and Response to Reviewers (without Author Details) an attention task that facilitates tests of these specific alerting, orienting, and executive components of attention in healthy subjects. The primary difference between the studies was the stimulation protocol used.”

3. The presentation of the "most commonly used forms" of TMS in research (2nd page of the section "Transcranial Magnetic Stimulation (TMS) in the Study of Attention") is not clear. For instance, "classic" repetitive TMS (rTMS) protocols do not entail bursts of stimuli, but "homogeneous" stimulation frequencies (as then mentioned in the following). Moreover, cTBS and iTBS are particular forms of rTMS (on a side note, references to work of the group that developed cTBS and iTBS are missing).

● We greatly appreciate the reviewer for finding this mistake. We have reevaluated this section detailing the different forms and protocols of TMS and have made the necessary changes to make our statements accurate. On pages 22 and 23, we fixed our error and wrote: “There are several types of TMS that can be applied in research. The two most used forms are repetitive stimulation and theta-burst stimulation. Repetitive TMS (rTMS) involves sending repeating pulses of a particular frequency to the scalp and the stimulation effects are dependent on the frequency. Low frequency, typically 1Hz, is considered to be inhibitory while 10Hz and above is considered to be excitatory. Another common form of stimulation is theta-burst stimulation (TBS) which is a protocol that was derived from rTMS where researchers were able to send repeated application of bursts of pulses to the human motor cortex [66]. TBS is thought to enact stimulation protocols similar to those seen in animal models to induce long term potentiation or depression [65]. Therefore, TBS involves sending patterns of short bursts at high frequencies and can be given in continuous or intermittent protocols. Continuous theta-burst stimulation (cTBS) is believed to be an inhibitory protocol with stimulation effects reflecting a long-term depression-like decrease in neural activity. Intermittent theta-burst stimulation (iTBS) is believed to be an excitatory protocol with stimulation effects reflecting long-term potentiation-like increases in neural activity..” Additionally, we cited the group who first used theta burst on the human motor cortex: Huang, Edwards, Rounis, Bhatia, & Rothwell, 2005.

4. Similar approaches as the one proposed in the review have already been postulated (e.g., Fox, 2018. Mapping symptoms to brain networks with the human connectome. New England Journal of

Medicine, 379(23)) or applied (e.g., Ozdemir et al., 2020. Individualized perturbation of the human connectome reveals reproducible biomarkers of network dynamics relevant to cognition. Proceedings of the National Academy of Sciences, 117(14)). The authors may want to follow up on this literature.

● We thank the reviewer for pointing to this research and we agree that it is necessary to address that we aren't the first to suggest an integrative framework for studying cognitive phenomena. On page 36 under the Integrating Methods for Precision Modulation and Cognitive Discovery, we wrote, "Similar integrative methods have been suggested previously, such as combining lesion analyses and the human connectome to create lesion network mapping to improve treatment targets [46], which indicates that multimodal approaches can improve research and research outcomes in various disciplines. Crucially, there has been some recent empirical evidence for an integrative model similar to what we are suggesting for cognitive discovery. It has been shown through the combination of fMRI guided, individualized TMS, simultaneous EEG, and reliable empirical paradigms that discrete neural networks can be perturbed and measured, and that those measurements can act as biomarkers for cognitive performance [104]. The results from this study can provide us with necessary information about the organization and role of specific neural networks in cognitive and behavior, which can then later better inform our treatment plans and patient outcomes." We still believe our review is an important addition to this line of literature as it specifically addresses attention mechanisms with a particular set of integrative methods but we are glad to acknowledge others in this field who have also identified the benefit of integrating advanced research methods for cognitive discovery.

5. A perturbation of a given network node by means of TMS has been shown to elicit compensatory processes in other distant nodes (see, e.g.: Sack AT et al., 2005. The dynamics of interhemispheric compensatory processes in mental imagery. Science 308; O'Shea J et al., 2007. Functionally specific reorganization in human premotor cortex. Neuron 54; Hartwigsen et al., 2013. Perturbation of the left inferior frontal gyrus triggers adaptive plasticity in the right homologous area during speech production. Proc Natl Acad Sci U S A 110). The Authors may want to follow up on this aspect.

● We are thankful to the reviewer for suggesting this research and we have included this literature in our paper. We think that readers will benefit from understanding how TMS has been used to study the cortex and cognition, as well as how TMS can influence neuronal activity directly. We believe that this may also bolster our own suggestion for integrating TMS with other methods to study cognition and behavior and to treat clinical deficits because this literature provides evidence for positive effects of TMS application, like plasticity following injury. On page 22 we wrote, "Additionally, direct evidence for TMS influencing cortical reorganization and neural plasticity has been found. Applying TMS to induce a temporary lesion to a distinct network in one hemisphere can result in immediate compensation by the contralesional hemisphere to take over the lesioned networks functions [126]. Furthermore, compensatory increases in the right premotor cortex and medial premotor areas have been observed, even without explicit behavioral effects following inhibitory TMS applied to the left premotor cortex [103]. Finally, plasticity for aphasia recovery was observed in the right inferior frontal gyrus following inhibitory stimulation with TMS to the left homologous cortical area [58]. Thus, TMS is useful because it has the capability to further explain how networks are organized, what their disparate functions may be, and how these networks can be plasticly altered following perturbations. With all of these uses and effects in mind, it can easily be seen that by targeting specific networks with TMS, a stronger causal link between neurons embedded within larger scale networks and behavior may be revealed."

6. The review focuses on results stemming from fMRI research. However, several studies combining

TMS and EEG have also shown to be able to deliver important information on the human connectome with high temporal resolution (see, e.g., for a review: Bortoletto et al., 2015. The contribution of TMS-EEG coregistration in the exploration of the human cortical connectome. Neurosci Biobehav Rev, 49). The Authors may want to integrate this perspective and/or comment on why to focus their perspective only on fMRI.

● We agree that these other research themes are very important; however for the sake of simplicity in this review, we focused on TMS-fMRI research. By focusing on fMRI research in this review, we are identifying opportunities for brain stimulation applications in the context of fast-emerging personalized techniques. With your comment in mind, we have added a section entitled "Complementary Approaches - TMS, EEG, and fMRI" on page 32 where we very briefly touch upon other anatomical and functional methods that could be implemented to enhance singlesubject neuromodulation.

● On pages 32 and 33, we wrote, "While we have emphasized using personalized fMRI mapping, it is important to remark upon other anatomical and functional methods that could enhance singlesubject neuromodulation.

In addition to TMS-fMRI integrated studies, TMS has been combined with EEG and has been important in uncovering temporally precise information regarding the human connectome. Specifically, integrating these techniques may allow researchers to better understand the brain rhythms involved in cognition and behavior through direct manipulation of cortical frequencies with TMS and temporally precise recordings with EEG [146]. TMS-EEG has also been helpful in assessing neural networks, revealing information about cortical excitability, effective connectivity, and oscillatory tuning of different brain areas [124]. This set of methods also has great clinical utility in identifying pathophysiological biomarkers for psychiatric and neurological disorders and in predicting treatment response [149]. Recently, investigators have achieved closed-loop protocols with TMS and EEG, where TMS pulses are triggered by a given EEG signal which may improve stimulation effects [97, 138].

Combining TMS, EEG, and fMRI has also been implemented to identify state-dependent effects and cause time-dependent activation. This multimodal application in research has allowed for direct neural manipulations while measuring spatially precise brain activity in response as well as measuring temporally precise fluctuations in cortical oscillations [49, 108]."

7. The Authors consider virtually only functional connectivity. However, already seminal papers have shown that also structural connectivity (in terms of properties of white matters tracts connecting attention networks nodes) plays a central role in individual variations in attentional performance (e.g., Thiebaut de Schotten et al., 2011. A lateralized brain network for visuospatial attention. Nat Neurosci 14(10)). Moreover, these individual differences in structural connectivity also modulate the effects of TMS on attentional performance (e.g., Chechlacz et al., 2015. Structural Organization of the Corpus Callosum Predicts Attentional Shifts after Continuous Theta Burst Stimulation. J Neurosci 35(46); Cazzoli et al., 2017. A matter of hand: Causal links between hand dominance, structural organization of fronto-parietal attention networks, and variability in behavioural responses to transcranial magnetic stimulation. Cortex 86; Schintu et al., 2020. Callosal anisotropy predicts attentional network changes after parietal inhibitory stimulation. Neuroimage 226). The Authors may want to integrate this perspective in their review.

● We appreciate the reviewer identifying this important area of study, which also identifies a crucial direction and opportunity to fuse anatomical and functional methods to enhance outcomes. We now state on pages 20 and 21 that "***Anatomical Connectivity***. Although functional imaging has been the

primary focus of this review, it is imperative to mention how structural imaging and anatomical connectivity has played a role in revealing cognitive capabilities. For example, tractography imaging has revealed that anatomical variability, distribution, and volume of white matter fibers can predict the lateralization of visuospatial attention [35]. Moreover, individual differences in the various white matter connections involved in visual attention processes are able to predict response to TMS [19, 20, 134]. While we focus on the importance of clarifying the role of individualized functional networks, the joint contributions of anatomy and function will be an important frontier in neuromodulation research."

8. It seems somehow odd to report only on ADHD as a model for the application of TMS in attention disorders. For instance, the Authors previously describe several empirical paradigms aimed at specifically assessing discrete aspects of attention in healthy individuals, but for the clinical application they report on three ADHD studies in which TMS effects on attention were assessed by means of subjective measures such as self-report and questionnaires.

● We chose to focus on ADHD as the clinical population of interest because we can more directly focus on how researchers have attempted to utilize non-invasive brain stimulation to treat the major tenets of attention, given ADHD symptoms involve these specific deficits. We focused on a subset of studies in healthy subjects that provide specific insights with respect to the computationally-based measures of attention subcomponents, and focused on ADHD studies in which similar inferences could be examined. The edits to this section on pages 26 and 27 will hopefully clarify to the reviewer why we have decided not to expand our review to other clinical populations.

● We decided to change our "Clinical Subjects" section to "ADHD Subjects" to clarify our specific focus on ADHD in this review.

● We also agree with the reviewer that it may seem odd to report on these studies given patients were assessed with subjective measures; however, we take this as an opportunity to further support one of our broader points that more research needs to be conducted using reliable and precise objective measurements and tasks and study their relationship to subjective clinical measures. Given that there is very little research studying TMS effects in ADHD subjects and that these studies do not use precise, process-specific instruments to measure attention, there is a pressing need to integrate these methods in the study of clinical attention deficits. We also note that despite these studies using subjective measures, they are nonetheless imperative to review given they do use TMS in the attempt to alleviate ADHD symptoms and improve attention.

○ On pages 26 and 27 we wrote, "By only reviewing studies that have aimed to treat the attention deficits in ADHD subjects, we can more directly focus on how researchers have attempted to utilize non-invasive brain stimulation to treat the major tenets of attention, such as top-down processing. However, it is important to point out that unlike the studies involving TMS and healthy subjects, these studies utilized solely subjective measures to evaluate attention performance. While subjective measures are important for gaining firsthand experience information from the patient, well-defined, reliable, and robust tasks that objectively measure attention performance will be necessary for accurately measuring the stimulation effects on attention behavior. Additionally, this should provide support for the notion that more research with clinical subjects, like those with ADHD, should be conducted that utilize objective cognitive and behavioral tasks. Nonetheless, despite the lack of explicit behavioral tasks to measure attention, these studies are pertinent to this review, as they are the major studies that have used TMS to treat attention deficits."

Comments from Reviewer 2

In the submitted manuscript the authors present an in-depth review of the neuroscience of attention in the context of extensive theoretical and empirical work, with a special emphasis on network neuroscience and non-invasive brain stimulation. Overall, this is an excellent review of the literature, and has the basis of many future empirical investigations. I only have a few conceptual issues that I would like the authors to address.

● We thank the reviewer for many helpful comments. We hope that our responses and edits have improved the clarity and rigor of this manuscript and would be happy to integrate any further suggestions.

1. The first 5 pages provide a nice review of the current state of knowledge. However, I feel this is a bit distracting from the purpose of the review, which in my opinion, starts at Brain Networks and Attention. I am sure there are several other review articles and book chapters that have summarized historical, theoretical, and empirical perspectives of attention, and perhaps these sections can be summarized more concisely in order to get to the main substance of the review article.

● We are grateful that the reviewer brought this to our attention and we agree that we do not need to go in depth on the attention background as there are many reviews on attention and its mechanisms for readers to reference. In response, we have condensed the first 5 pages to include only the most pertinent background information. We do still want to address the evolution of the study of attention while citing seminal studies in order to focus on one of the main ideas, which is that as we acquire, converge, and use more advanced and precise methods, we can move towards a better understanding of cognitive phenomena. In addition, we cut down the major themes of the attention section while keeping the main definitions and ideas so that all readers can comprehend the language and concepts that we use throughout the remainder of the paper. History and background is now pages 2-5.

2. The author details the conventional methods of applying TMS (iTBS, cTBS, 10hz rTMS and 1hz-rTMS). However, the excitatory/inhibitory effects of the protocol are largely based on motor cortex studies, particularly TBS. I think the author should discuss the stimulation effects of these protocol beyond the motor cortex, but more of the observed effects of these protocol on other brain regions, which sometime are incompatible with the dogma of these motor cortex studies.

● We agree with the reviewer that we should include literature that has aimed to validate stimulation effects in other cortical areas besides the motor cortex, especially given that our focus is on TMS effects on cognitive and associative areas. We now include on page 23, "These are the most conventional stimulation sequences applied in research and clinically, and their effects were validated in studies of the motor cortex using outcome measures like motor-evoked potentials [36, 87, 100]. The application of these protocols to other areas of the human cortex thought to mediate cognition is our focus, and therefore it is crucial to discuss stimulation effects beyond the motor cortex. However, due to the lack of clear, temporally fast outcome measures in other cortical areas, it has been difficult to obtain valid and reliable markers of inhibitory and excitatory activity, leading investigators to principally use these sequences in research and clinical paradigms. Thus, there is a future need to obtain estimates of cortical and behavioral responses using these and other parameters. A more recent research avenue to

reconcile this issue is the use of combined TMS-EEG to obtain potential markers of excitatory and inhibitory activity [14, 40, 41]; this methodology will be further reviewed in a later section."

3. In regards to conventional TMS methods, other methods have also been employed to study attention (closed-loop TMS, combined EEG - TMS). Animal TMS studies may also provide additional information about the effects of TMS on attentional networks.

● On page 32, we included a new section entitled Complementary Methods - TMS, EEG, and fMRI to provide a very brief review of TMS-EGG, TMS-EEG-fMRI, and closed loop protocols. We believe this will provide readers with the opportunity to explore this literature if they are interested in these techniques.

4. There has been a few recent studies using simultaneous TMS and fMRI to study brain networks (resting state). Perhaps adding these to the review is relevant here.

● We appreciate the reviewer suggesting this set of literature to us and we agree that adding them to our review is relevant. On pages 30 and 31, we wrote, "These parcellations could be used in traditional test, stimulate, and test paradigms (e.g., before and after the subject enters the scanner), or simultaneously with fMRI. For instance, to test the mechanism of action of TMS application to the dorsolateral prefrontal cortex in major depressive disorder treatment, researchers used TMS-fMRI to reveal the propagation of induced neuronal activity associated with stimulation [159]. Concurrent TMS and fMRI is therefore useful to map neural circuits and uncover how brain regions communicate with one another, given its ability to measure spatially precise cortical and subcortical changes in activity following perturbation via stimulation [12, 101]."

5. In regards to network science, the author should also review the implications of different methodological approaches to identify brain networks and quantify their correlations in the context of brain networks and mental disorders

● We agree that we should review different approaches to identifying networks and on page 32 have now stated, "It should also be mentioned that there are multiple ways to define group and individualized parcellations, with investigators using network clustering techniques or independent component analysis: (c.f., Salehi, Karbasi, Scheinost, & Constable [131]; Salehi, Karbasi, Shen, Scheinost, & Constable [132]; Salehi, Karbasi, Barron, Scheinost, & Constable [130]; Han et al. [56]; and Chong et al. [23]). Validating such parcellations and their role in cognitive functions with multiple methods including TMS or other neural stimulation and recording techniques in individual subjects is an important direction for future research. Furthermore, the reliability and validity of these methods in clinical samples must be evaluated, and the predictive validity to clinical status and TMS outcomes should be tested."

2nd Decision letter

Manuscript No.: CRNEUR-D-20-00014R1
Title: The Role of Transcranial Magnetic Stimulation in Understanding Attention-Related Networks in Single Subjects
Journal: Current Research in Neurobiology

Dear Ms Yeager,

Thank you for submitting your manuscript to Current Research in Neurobiology.

I have completed my evaluation of your manuscript. The reviewers recommend reconsideration of your manuscript following minor revision and modification. I invite you to resubmit your manuscript after addressing the comments below. Please resubmit your revised manuscript by Jul 20, 2021.

When revising your manuscript, please consider all issues mentioned in the reviewers' comments carefully: please outline every change made in response to their comments and provide suitable rebuttals for any comments not addressed. Please note that your revised submission may need to be re-reviewed.

Current Research in Neurobiology values your contribution and I look forward to receiving your revised manuscript.

CRNEUR aims to be a unique, community-led journal, as highlighted in the Editorial Introduction. As part of this vision, we will be regularly seeking input from the scientific community and encourage you and your co-authors to take the survey.

Kind regards,

Anna S Mitchell, Ph.D.
Editor in Chief
Current Research in Neurobiology

Comments from Editors and Reviewers:

Thank you, Brooke, for addressing all the reviewers comments. Please can you also address the one further point raised by Reviewer 1 and return the manuscript and I will go on to accept it for publication.

Reviewer #1:

I would like to thank the Authors for addressing my comments. I only have a further comment: the beginning of the section "Healthy subjects" on page 27 could still be somewhat confusing, starting with the sentence "There have been three studies which have tested the effects of TMS on attention in healthy subjects, all of which stimulated brain regions revealed by the activation-based literature...". As the Authors state later, there seems to be only three studies that assessed the effects of TMS on attention by means of the ANT paradigm in particular, and not generally "the effects of TMS on attention in healthy subjects". If that's the case, the Authors should probably state this more clearly from the beginning, justifying their choice of concentrating their reviewer on the ANT paradigm (and making sure that in the literature there are indeed only the three studies that they cite).

Reviewer #2:

I applaud the authors for their responses to my comments, as well as the comments of the other reviewer. I believe the research presented here will be a valuable resource for future research. I have no other comments to provide on the manuscript.

2nd Author Response Letter

Response to comments from Editors and Reviewers:

Comments from Reviewer 1

I would like to thank the Authors for addressing my comments. I only have a further comment: the beginning of the section "Healthy subjects" on page 27 could still be somewhat confusing, starting with the sentence "There have been three studies which have tested the effects of TMS on attention in healthy subjects, all of which stimulated brain regions revealed by the activation-based literature...". As the Authors state later, there seems to be only three studies that assessed the effects of TMS on attention by means of the ANT paradigm in particular, and not generally "the effects of TMS on attention in healthy subjects". If that's the case, the Authors should probably state this more clearly from the beginning, justifying their choice of concentrating their reviewer on the ANT paradigm (and making sure that in the literature there are indeed only the three studies that they cite).

●On pages 24 and 25 we wrote, Within this section, we will focus on three studies which have tested the effects of TMS on attention in healthy subjects, all of which stimulated brain regions revealed by the activation-based literature: Xu et al. [163], He et al. [61], and Xu et al. [164]. A commonality among these studies was that researchers measured attention with the Attention Network Test (ANT), which is a task that distinguishes between and measures the efficiencies of the three putative sub-systems of attention: alerting, orienting, and executive inhibition [42]. While there has been more research that has used TMS to study attention, we concentrate on these particular studies because the ANT was designed to focus on three well-established components of attention based on the Posner model that are associated with three specific different networks and behaviors [42. 43, 113]. Additionally, they include a TMS manipulation for relatively focal stimulation. Thus, pairing TMS and the ANT supports more specific tests of brain-behavior associations than tasks that do not separate these three processes and their corresponding circuits. We hope that this makes it clear that there are other studies that use TMS to study attention, but we chose to focus on these three studies given that they include the ANT and TMS manipulation. We specifically want to get our point across that the ANT separates and measures the efficiency of the three sub-systems of attention and that this may allow for more explicit and targeted associations to be made between brain networks and particular functions.

Accept Letter

Dear Ms Yeager,

Thank you for submitting your manuscript to Current Research in Neurobiology.

I am pleased to inform you that your manuscript has been accepted for publication.

My comments, and any reviewer comments, are below.

Your accepted manuscript will now be transferred to our production department. We will create a proof which you will be asked to check, and you will also be asked to complete a number of online forms required for publication. If we need additional information from you during the production process, we will contact you directly.

We appreciate and value your contribution to Current Research in Neurobiology. We regularly invite authors of recently published manuscript to participate in the peer review process. If you were not already part of the journal's reviewer pool, you have now been added to it. We look forward to your continued participation in our journal, and we hope you will consider us again for future submissions.

CRNEUR aims to be a unique, community-led journal, as highlighted in the Editorial Introduction. As part of this vision, we will be regularly seeking input from the scientific community and encourage you and your co-authors to take the survey.

Kind regards,

Anna S Mitchell, Ph.D.
Editor in Chief
Current Research in Neurobiology

Editor and Reviewer comments:

Thank you for addressing the final point raised by the reviewer.

-------- End of Review Comments --------
